# Design of Novel, Water Soluble and Highly Luminescent Europium Labels with Potential to Enhance Immunoassay Sensitivities

**DOI:** 10.3390/molecules22101807

**Published:** 2017-10-24

**Authors:** Henri Sund, Kaj Blomberg, Niko Meltola, Harri Takalo

**Affiliations:** 1 Radiometer Turku Oy, Biolinja 12, 20750 Turku, Finland; henri.sund@radiometer.fi (H.S.); kaj.blomberg@radiometer.fi (K.B.); niko.meltola@arcdia.com (N.M.); 2 Present affiliation ArcDia International Oy Ltd., Lemminkäisenkatu 32, 20520 Turku, Finland

**Keywords:** europium chelate, label, (phenylethynyl)pyridine chromophore, luminescence, intra-ligand charge transfer

## Abstract

To meet the continual demands of more-sensitive immunoassays, the synthesis of novel luminescent Eu(III) chelate labels having similar substituted 4-(phenylethynyl)pyridine chromophores in three different chelate structure classes are reported. Significantly enhanced luminescence intensities were obtained, evidently caused by the intra-ligand charge transfer (ILCT) mediated sensitization, but the alternative ligands triplet state process cannot be ruled out. Based on the present study, even quite small changes on the chelate structure, and, especially, on the substituents’ donor/acceptor strength on both ends of 4-(phenylethynyl)pyridine subunits have an unpredictable effect on the luminescence. The highest observed brightness was 16,400 M^−1 ^cm^−1^ in solution and 69,500 M^−1 ^cm^−1^ on dry surface, being 3.4 and 8.7 fold higher compared to the reference chelate. The new label chelates provide solutions for improved assay sensitivity up-to tenfold from the present concepts.

## 1. Introduction

Time-resolved fluorometry (TRF) employing long-lifetime emitting luminescent lanthanide chelates has been applied in many specific binding assays, such as immunoassays, DNA hybridization assays, receptor-binding assays, enzymatic assays, bio-imaging such as immunocytochemical, immunohisto-chemical assays or cell based assays to measure the wanted analyte at very low concentration [1,2,3]. Lanthanide chelates have special properties offering proper alternative markers in bio-affinity assays: (i) the difference between the excitation and emission wavelengths is large; (ii) normally, they have long emission life-time compared to the background, and, thus, the time-resolved fluorescence (TR-FIA) measurement techniques together with narrow emission lines eliminate the fluorescence background almost completely; (iii) the concentration quenching is small and enables using wide dynamic assay range without additional dilutions, and (iv) several different sample matrixes e.g., whole blood, serum and plasma can be used for the assays. Applications based on TRF have been commercialized in the fields of clinical diagnostics and drug discovery [1].

For TRF applications, an optimal label has to fulfill several strict requirements. First, the label has to be photo-chemically stable both in the ground and excited states, and it has to be kinetically and chemically stable. The excitation wavelength has to be as high as possible, preferable over 300 nm. It has to have efficient cation emission i.e., intense brightness (excitation coefficient x quantum yield, εΦ). The lanthanide chelate has to have a high absorptivity and efficient energy transfer from the excited level of the so-called antenna ligand (i.e., chromophore) to the proper emitting level of the lanthanide ion. Luminescence quenching by water molecules should be prevented to enable a long luminescence decay time, but, at the same time, the chelate has to have good water solubility. For the purpose of labeling, it should have a reactive group to allow covalent attachment to a bio-specific binding reactant, and the affinity and nonspecific binding properties of the labeled biomolecules have to be retained. It is challenging to prepare a chelate-label molecule that fulfills all requirements, and, therefore, certain compromises are generally made in the development of suitable labels. As a consequence hereof, a number of attempts (see e.g., [4]) have been made to tune the photo-physical properties of the chelate labels suitable for TRF applications, and only a few viable chelate labels are in commercial use.

**Scheme 1:**
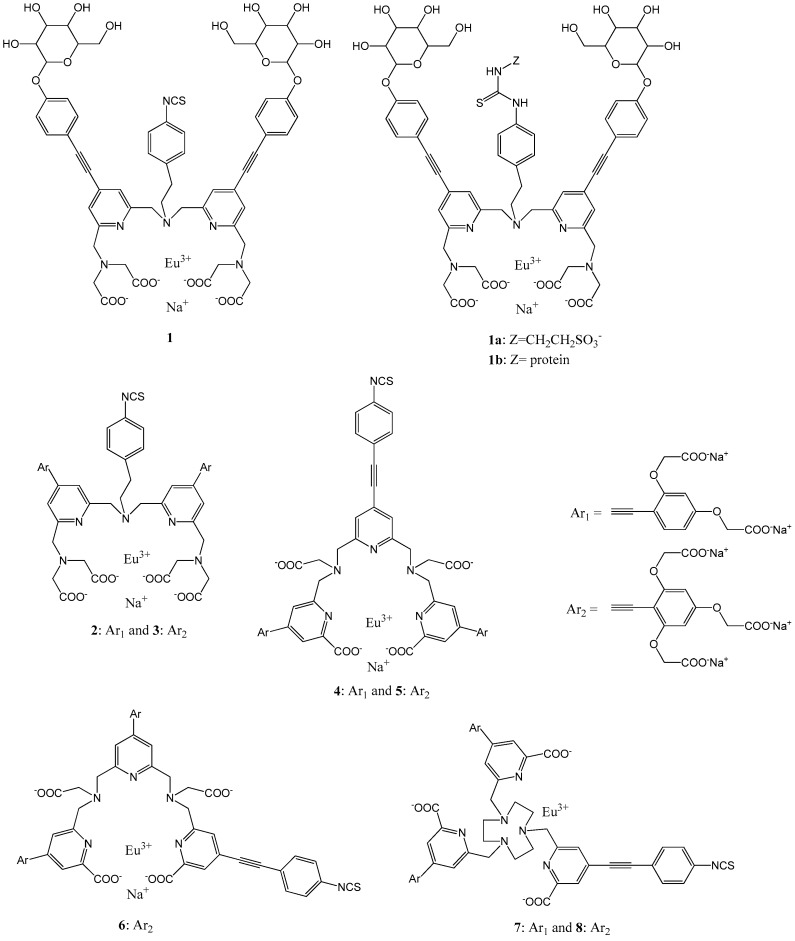
The studied label structures numbered **1**–**8**.

Since the discovery in the mid-1980s and the later publication of the phenylethynylpyridine antenna for Eu(III) chelates [5,6], several new chelates utilizing the chromophore have been described in literature, inter alia three [6,7,8,9,10], seven [6,7,8,11,12,13] and nine-dentate acyclic [8,14,15] as well as macro-cyclic ligands [8,16,17,18,19]. The nine-dentate Eu(III) label (**1** in Scheme 1) has been applied already almost for 15 years in TRF immunoassays [14,20,21,22,23,24] especially in near-patient testing such as critical care and emergency situations. The technology has offered quick, easy-to-use, high quality and quantitative point-of-care testing platform called as *AQT90 FLEX* (*Radiometer Medical ApS*). The aim of the present work is to enhance the brightness and to red-shift the excitation wavelength of the present label **1** to be able to use a UV LED based excitation [25] with significantly improved assay sensitivity. For that purpose, we prepared three different nine-dentate chelate label designs i.e., with two and three individual chromophore moieties in acyclic and macrocyclic ligand formats (chelates **2**–**8** in Scheme 1). The objective was to study the effect of electron-releasing substituents (OCH_2_COO^−^) in mesomeric *para*- and *orto*-position of phenylethynylpyridine moiety together with electron-withdrawing (COO^−^) as well as releasing (CH_2_) groups at the other end of the chromophoric moiety in those three different ligand groups. The novel chelates were tested after coupled with taurine (**2a**–**8a**) and protein (**2b**–**8b**) being comparable to the derivative **1a**,**b** of the used reference chelate **1**.

## 2. Results

### 2.1. Syntheses

**Scheme 2:**
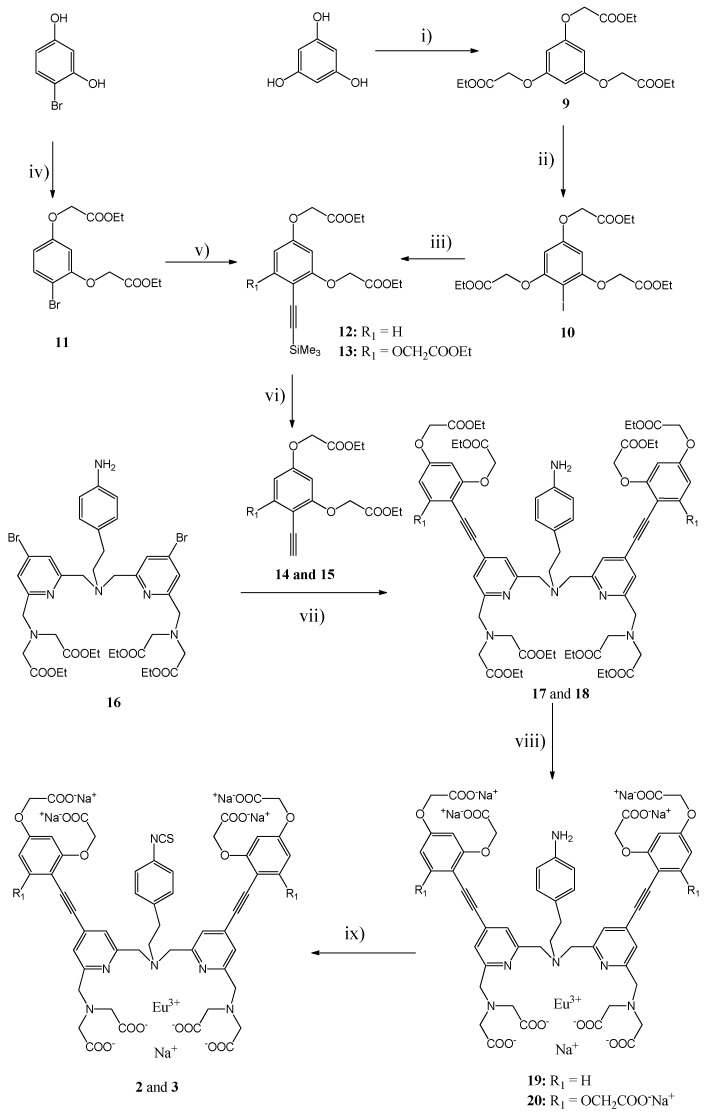
Synthesis of compounds **2** and **3**. *Reactions*: (i) and (iv) BrCH_2_COOEt, K_2_CO_3_, MeCN; (ii) NCS, NaI, CH_3_COOH; (iii) and (v) trimethylsilylacetylene (TMSA), Pd(II), CuI, DEA, DMF; with the compound **11** PPh_3_ was used as an additional catalyst; (vi) TBAF, CH_2_Cl_2_; (vii) Pd(II), CuI, TEA, THF; (viii) KOH, EtOH, H_2_O, EuCl_3_; (ix) CSCl_2_, NaHCO_3_, CHCl_3_, H_2_O.

The syntheses of two different terminal acetylenes **14** and **15** used in the study started from 4-bromobenzene-1,3-diol and benzene-1,3,5-triol, which were transformed to the corresponding intermediates **11** and **9** with the reaction of ethyl bromoacetate in dry MeCN and using K_2_CO_3_ as the needed base (Scheme 2). For the synthesis of compound **9,** careful control of reaction conditions is required to achieve a reasonable yield; otherwise, a dramatic drop in yield together with purification problems will be the result. Direct iodination of the compound **9** with I_2_ and NaHCO_3_ in H_2_O/CHCl_3_ mixture afforded poor results, whereas the reaction with *N*-chlorosuccimide and NaI in acetic acid described by Yamamoto et al. [26] produced the iodo derivative **10** in quantitative yield. As an alternative route to the compound **10**, a reaction of 2-iodo-benzene-1,3,5-triol [27] with ethyl bromoacetate was entirely unsuccessful. Both halides **10** and **11** were conjugated with trimethysilylacetylene using the Sonogashira reaction in the presence of a catalytic amount of Pd(II) catalyst and CuI under argon. Microwave heating at 100 °C for 30–45 min in diethyl amine (DEA) and dimethylformamide (DMF) gave appropriate yields of products. In the reaction with **11,** an improved yield was obtained by using PPh_3_ in the mixture. We also tested using a corresponding bromo derivative to the compound **10**—nicely obtained by the reaction of **9** with *N*-bromosuccimide, but, unfortunately, without success. Finally, the deprotection with tetrabutylammonium fluoride gave the wanted phenylacetylenes **14** and **15** with quantitative yields. 

The second Sonogashira reaction between the acetylenes **14** and **15** and the dibromo intermediate **16** [14] gave ligand esters **17** and **18**, respectively. The esters **17** and **18** were hydrolyzed with KOH in EtOH–H_2_O followed by the forming of chelates **19** and **20** with EuCl_3_ in slightly acidic conditions and precipitating the excess europium as Eu(OH)_3_ by adjusting the pH to 8.5. The chelates **19** and **20** were purified by the semi-preparative HPLC.

The activation of the amino group of the chelates by transformation to the corresponding isothiocyanato group in the final labeling reagents **2** and **3** was performed with thiophosgene in H_2_O/CHCl_3_.

**Scheme 3:**
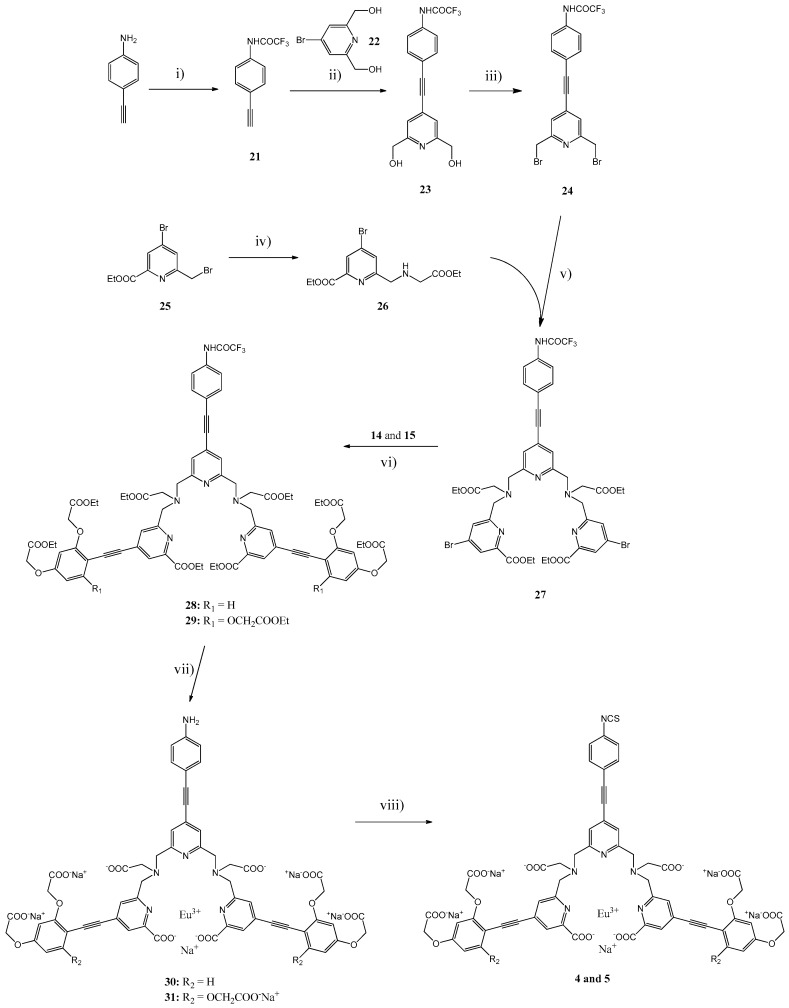
Synthesis of compounds **4** and **5**. *Reactions*: (i) TFAA; (ii) Pd(II), CuI, triethylamine (TEA), tetrahydrofuran (THF); (iii) PBr_3_, CHCl_3_; (iv) NH_2_CH_2_COOEt, N,N-diisopropylethylamine (DIPEA), MeCN; (v) K_2_CO_3_, MeCN; (vi) Pd(II), CuI, TEA, DMF; (vii) KOH, EtOH, H_2_O, EuCl_3_; (viii) CSCl_2_, NaHCO_3_, CHCl_3_, H_2_O.

The synthesis of the acyclic labeling regents (**4** and **5** in Scheme 3) with three chromophores started from the protection of aminophenyl acetylene with trifluoroacetic acid anhydride to get compound **21**. After the Sonagashira coupling reaction with the 6-bromo-2,6-di-hydroxy-methylpy-ridine **22** [28], diol **23** was transformed with PBr_3_ into bis(bromomethyl) derivative **24**. This product was conjugated with two pyridine subunits **26** in the dry MeCN in the presence of K_2_CO_3_ to get the key intermediate **27**. The needed building block **26** was prepared from 4-bromo-6-bromomethyl-2-carboxyethylpyridine **25** [14] using five-fold excess of glycine ethyl ester hydrochloride in dry MeCN and di-isopropylethylamine. After the triplet bond coupling reaction of the two bromo groups in the compound **27** with the acetylenes **14** and **15**, the target ester ligands **28** and **29** were hydrolyzed, transformed to the corresponding Eu(III) chelates **30** and **31** including the HPLC purifications, and, finally, activated to the target labeling chelates **4** and **5** as described above with the chelates **2** and **3**.

**Scheme 4:**
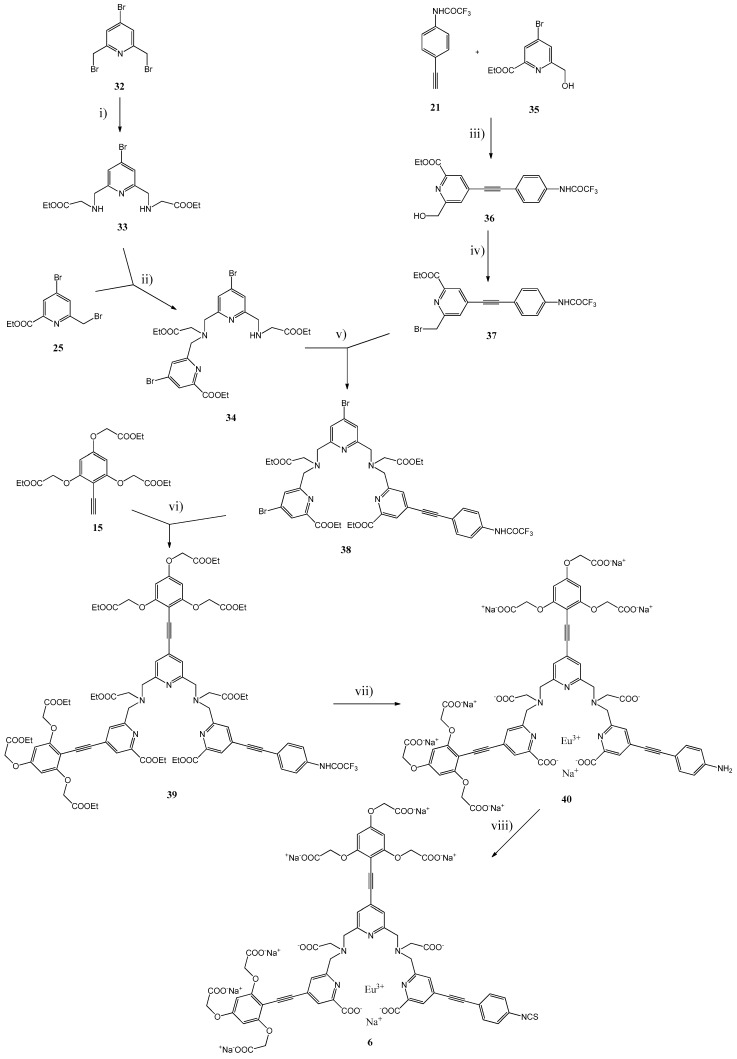
Synthesis of compound **6**. *Reactions*: (i) NH_2_CH_2_COOEt, DIPEA, MeCN; (ii) and (v) K_2_CO_3_, MeCN; (iii) and (vi) Pd(II), CuI, TEA, THF; (iv) PBr_3_, CHCl_3_; (vii) KOH, EtOH, H_2_O, EuCl_3_; (viii) CSCl_2_, NaHCO_3_, CHCl_3_, H_2_O.

The synthesis of the unsymmetrical acyclic analogue to the labeling reagent **5** i.e., the Eu(III) chelate **6** in Scheme 4 was more complicated. First, the 6-bromo-2,6-dibromomethylpyridine **32** [28] was transformed to diester **33** by the reaction with two ethyl glysinates, and, after conjugation to one equivalent of the compound **25**, the triester **34** was obtained with a reasonable yield. It is worth mentioning that, although in the last step only one main product spot is seen on TLC, besides the wanted product **34**, the TLC spot actually also contains a product in which two equivalents of the compound **25** have reacted with the compound **33**. With a careful column chromatography, the two products can be separated from each other. The secondary amino in **33** was coupled to the bromomethyl group of **37** in almost quantitative yield. The coupling reaction between acetylene **21** and 4-bromo-6-hydroxymethyl-2-carboxyethylpyridine **35** [14] followed by the bromination of the hydroxyl group in **36** with PBr_3_ gave the compound **37**. After a similar Sonagashira reaction as described above, the ligand ester **39** was formed followed by the hydrolysis, the formation of Eu(III) chelate **40**, the HPLC purification and activation to the labeling reagent **6**.

**Scheme 5:**
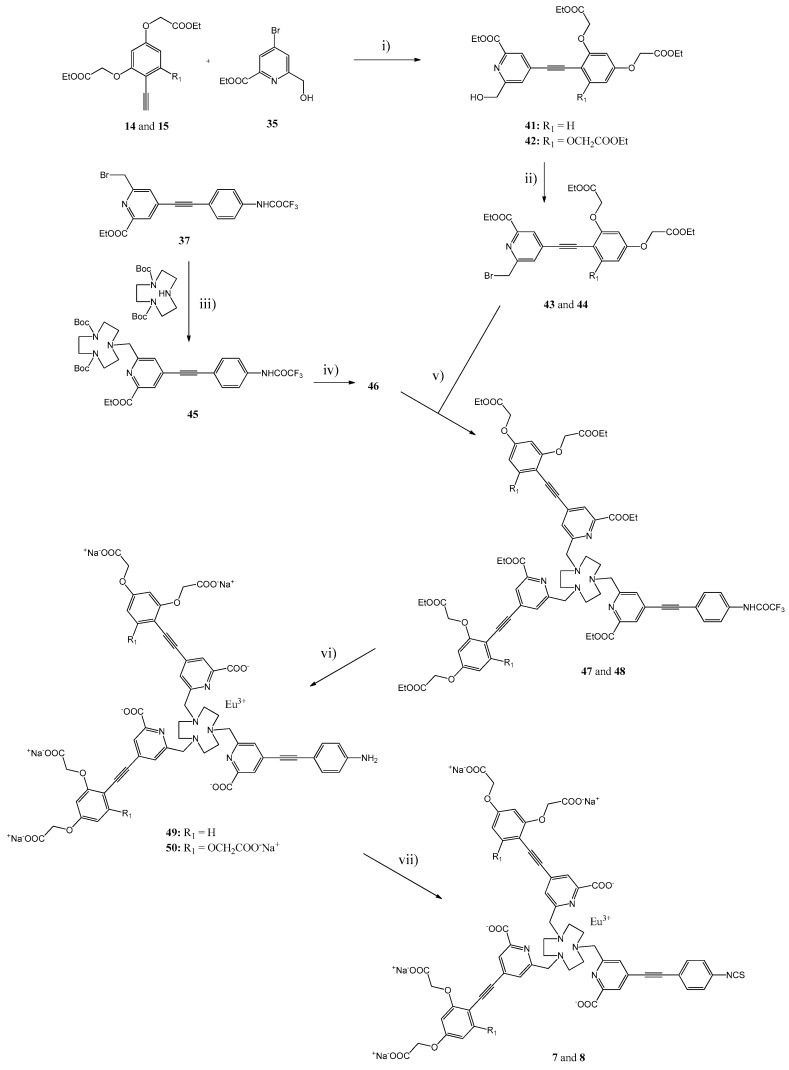
Synthesis of compounds **7** and **8**. *Reactions*: (i) Pd(II), CuI, TEA, THF; (ii) PBr_3_, CHCl_3_; (iii) K_2_CO_3_, MeCN; (iv) TFA; (v) DIPEA, MeCN; (vi) KOH, EtOH, H_2_O, EuCl_3_, citric acid; (vii) CSCl_2_, NaHCO_3_, CHCl_3_, H_2_O.

The synthesis of macrocyclic Eu(III) chelates **7** and **8** is presented in Scheme 5. After the organometallic coupling of the acetylenes **14** and **15** with the 4-bromo-6-hydroxymethyl-2-carboxy-ethylpyridine **35** to get the conjugates **41** and **42,** and the bromination of the hydroxyl group with PBr_3_, the needed chromophores **43** and **44** were isolated to be used for coupling with two amino functions of the macrocyclic intermediate **46**. The diBoc-protected 1,4,7-triazacyclononane derivative **45** formed from **37** and di-tert-butyl 1,4,7-triazacyclononane-1,4-dicarboxylate (diBocTACN) was deprotected with CF_3_COOH to get the compound **46**. The two phenylethynylpyridine subunits **43** and **44** were coupled with **46** in dry MeCN and DIPEA with high yields. It is notable that, according to mass spectra analysis of the crude reaction mixture, a mass corresponding of an over conjugated product was found i.e., the reactivity of bromide in **43** and **44** is high enough to be able to form ternary substituted amino function, although steric hindrance could be assumed significant enough to prevent such a reaction. The synthesis strategy to prepare the chromophores before conjugation to the 1,4,7-triazacyclononane (TACN) was chosen as the organometallic coupling reaction was not efficient when TACN was present, probably due to possible complex formations between Cu(I)/Pd(II) and the azacrown, which lead to incomplete reaction.

The ethyl ester groups of **47** and **48** were saponified with KOH in EtOH–H_2_O mixture; however, the conventional Eu(III) chelate formation in slightly acidic solution at room temperature lead to incomplete chelate formation according to HPLC and MS analyses. After multiple tests, the reasonable complex formation of **49** and **50** was achieved within several days incubation at 95 °C and pH 9.5 in aqueous citric solution to prevent Eu(OH)_3_ precipitation. The possible reason for the slow complexation could be the high negative charge of the two chromophores causing repulsion and slow rotation of the chromophores to reach the final complex i.e., high rigidity of the ligand and/or a possible strong encapsulation of potassium ion in the TACN cavity during the earlier phase, as has been seen with e.g., crown ethers and cryptands. After the HPLC purification of chelates **49** and **50**, the amino groups were activated with thiophosgene to receive the labeling reagents **7** and **8**.

### 2.2. Photophysical Properties

The reference chelate (**1**) and prepared labeling reagents (**2**–**8**) were conjugated to taurine (**→1a**–**8a**) and protein (**→1b**–**8b**) followed by purifications. The photochemical properties i.e., the absorption maxima (λ_abs_), the excitation maxima (λ_exc_), decay-times (τ), the corresponding molar absorption coefficients (ε), brightness (εΦ) and quantum yield (Φ) in tris-saline-azide (TRIS) buffer (TRIS 50 mM, NaCl 0.9%, pH 7.75) are presented in Table 1. The table includes also the obtained enhanced signals from dried troponin I immunoassay wells, compared with the corresponding signal of the reference chelate with a known effect of drying [20].

The excitation wavelengths of the new chelates are ca. 15–20 nm red-shifted compared to the reference chelate, and are between 340–350 nm due to the additional electron-releasing ether functions at *para*- and *meta*-positions of the chromophores. The excitation maxima fit perfectly to a recently published new optical system based on UV light emitting diode excitation at 340 nm [25]. The third ether substituent (e.g., chelate **5** compared to **4**) does not have any notable effect on the excitation wavelength, except with the chelate **2** (ca. 345 nm) compared to the corresponding complex **3** (ca. 350 nm). Generally, the increase of the chromophores decreases the H_2_O solubility as well as increases the formation of aggregates during the bio-molecule labeling process and non-specific binding properties for the labelled proteins. Aggregations will produce purification problems and reduce the yield of labelled material. Moreover, increased non-specific binding of the labelled biomolecule can enhance the assay background, and, thus, reduces the assay sensitivity. In the present study, the additional COO^−^Na^+^ groups, together with the ether substituents offer high solubility in H_2_O, reduce the nonspecific binding of the labelled biomolecule, and the affinity properties of the used biomolecules are almost unchanged despite the increased negative charge of the labels. However, such groups close to each other might cause nonspecific binging to positive groups present in the assay, and, thus, interference with assay components might be obtained. 

The absorptivities between the two chromophore chelates **1a**–**3a** are close to each other from 55,000 to 58,000 M^−1 ^cm^−1^. As it could be assumed, the third chromophore increases the absorptivities. The measured values from 64,000 (**5a**) to 89,000 M^−1^ cm^−1^ (**7a**) express the higher value for the 2,4-substituted taurine chelate designs compared to the corresponding 2,4,6-substitued ones. The coupling of chelate to the protein changes the polarity of the chelates environment and seems to enhance absorptivity with all new chelates except **4b** and **6b**, and the change is strongest with the TACN based label **8b** (104,000 vs. 77,000 M^−1 ^cm^−1^). 

The luminescence decay-times of the new chelates with two chromophores **2** and **3** are slightly shorter compared to **1**, and are more reduced with the acyclic chelates with three chromophores **4**–**6** being lowest with TACN derivatives **7** and **8**. Moreover, the life-times with 2,4,6-trisubstituted chelate designs are shorter compared to the corresponding 2,4-disubstitued analogues (**3** vs. **2**, **5** vs. **4** and **8** vs. **7**). Moreover, the chromophore with an electron donating NH(C=S)NH group can have its own influence on the luminescence lifetime. This phenomenon of luminescence lifetime reduction is rather surprising as all chelate structures are nine-dentate and the chromophores are similar. Generally, the sensitization of lanthanide luminescence occurs through the ligands’ triplet excited state. However, lately, an alternative route through an intra-ligand charge transfer (ILCT) state has been demonstrated [10,29]. Ligands, which contain a conjugated π-electron skeleton, such 4-(phenylethynyl)pyridine moiety, substituted by an electron-donating substituent together with an electron accepting substituent at the other end of the antenna, can be excited through the ligands’s CT-state. Such antenna chromophore can present a dipolar (push-pull) geometry. The lanthanide ion acting as a Lewis acid increases the acceptor strength of this push–pull feature and lowers the energy of the relaxed CT excited state. When the CT state is low enough, the excited energy can transfer back from the excited state of lanthanide ion, which is observed as a reduced luminescence life-time. Thus, the shortened luminescence life-time can be assumed to reflect the energy transfer through the ILCT together with the original triplet state mediating route. It is known that the relaxation in the CT excitation path is strongly correlated with the donor/acceptor pair, which supports the shorter luminescence life-time of the chelates having chromophores with only one CH_2_ group and one electron accepting COO^−^ group in the pyridines (**7** and **8**) over the other chelates having chromophores also with two CH_2_ groups. Thus, the chelates **7** and **8** have chromophores with lower CT states, whereas the others have subunits with CT state(s) at a higher energy level, which minimized both the energy transfer trough ILCT and the energy backflow. Similarly, the complexes with two chromophores **1**–**3** have only electron donating CH_2_ groups in pyridine and show the longest lifetimes. Moreover, reference chelate **1** has only one electron donating substituent in the phenyl ring compared to others, and, thus, the longest decay time is observed. As a consequence, the increase of luminescence lifetime in order 1 > 2 > 3 > 4 > 5 = 6 > 7 > 8 can be attributed to the push–pull feature of the complex chromophores. Further evidence for the energy back-transfer phenomenon due to the low lying ILCT state indicates the luminescence lifetime measurements of chelates **6b** (0.55 ms) and **8b** (0.42 ms) in D_2_O, which rule out the possible lifetime reduction caused by water molecules.

**Figure 1:**
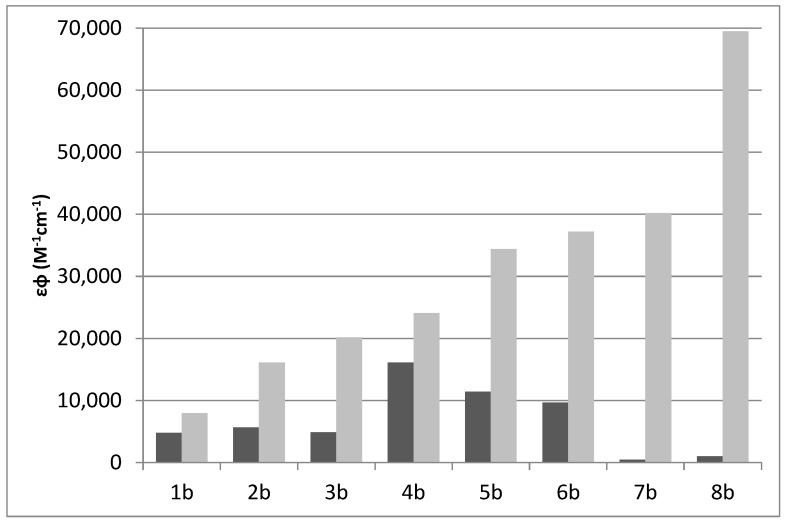
Brightness of the chelates **1b**–**8b** in 0.05 M TRIS buffer (dark grey) and on a dried surface (light grey).

**Figure 2:**
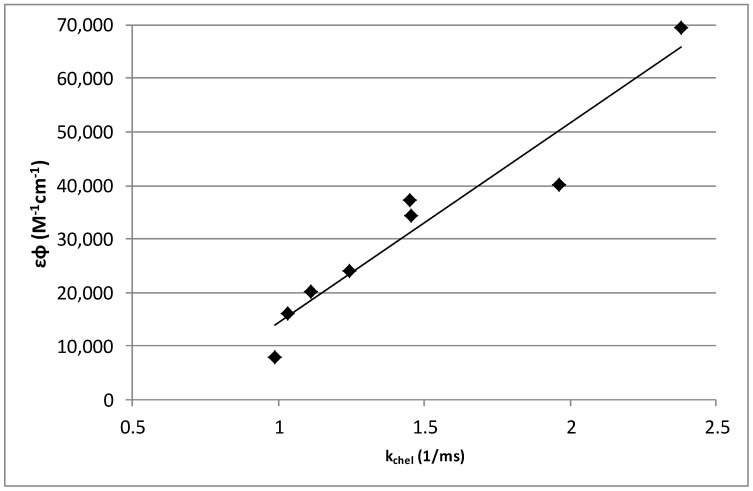
Measured enhanced brightness of chelates **1b**–**8b** on the dried surface vs. decay constant (k_chel_ = 1/τ) measured in 0.05 M TRIS buffer.

The luminescence effectiveness of the chelates is affected by the molar absorption coefficient (ε) and the quantum yield (Φ), and it can be illustrated by the product εΦ, called brightness. From the chelates with two chromophores **2a** gives almost two fold higher signal, whereas **3** is about at the same level compared to reference **1**. The acyclic chelates **4**–**6** show notable enhanced luminescence intensities (9700–16,400 M^−1 ^cm^−1^), being 2.0–3.1 times higher compared to the chelate **1**. In addition, the quantum yields (13–26%) are significantly increased. A remarkable drop of brightness and quantum yield is seen with the chelates **7** and **8**. The reason behind the obtained luminescence intensities cannot be explained by differences between triplet state energy levels as those can be assumed to be near to each other. Earlier triplet state energy level measurements from several different chelate designs having the same (phenylethynyl)pyridine subunit including three, seven and nine-dentate chelates have given the triplet state energy values in a range of 21,600–21,830 cm^−1^ [8]. Therefore, the observed effect on the luminescence intensity should be attributed to the excited energy transfer through the low lying ILCT states, although the triplet mediated process cannot be entirely ruled out. If the ILCT state is too low, the signal is reduced in aqueous solutions. Further evidence of the energy transfer through the ILCT state is seen from the brightness measurements on the surface dried assay wells after an immunoreaction (Table 1) compared to the corresponding wet measurements. The signal increase of 60% (i.e., 1.7 fold) for the chelate **1** has been demonstrated in literature [20]. It is known that with lanthanide chelates, which have low-lying CT state, the signal is highly dependent on solvent or environment polarity [29]. The enhanced luminescence on the dried surface are without doubt partly caused by the loss of H_2_O molecules causing the signal reduction due to the IR overtones of OH bonds in aqueous solution but also by the polarity change. The signal enhancement between wet and dry formats is ca. 2.8–4.1, 1.5–3.8 and 69–80 folds for the two chromophores containing chelates **2** and **3**, the acyclic chelates **4**–**6** and the TACN chelates **7** and **8**, respectively (see also Figure 1). When compared to the dry signal level of chelate **1**, brightness improvement from 2.0 to 8.7 fold is obtained. More evidence of the CT route and its significance on the luminescence is clearly seen from Figure 2. A quite clear correlation is seen between the decay constants (k_chel_ = 1/τ) in TRIS buffer and the measured enhanced brightness of the different chelates (**1b**–**8b**) after the drying.

**Figure 3:**
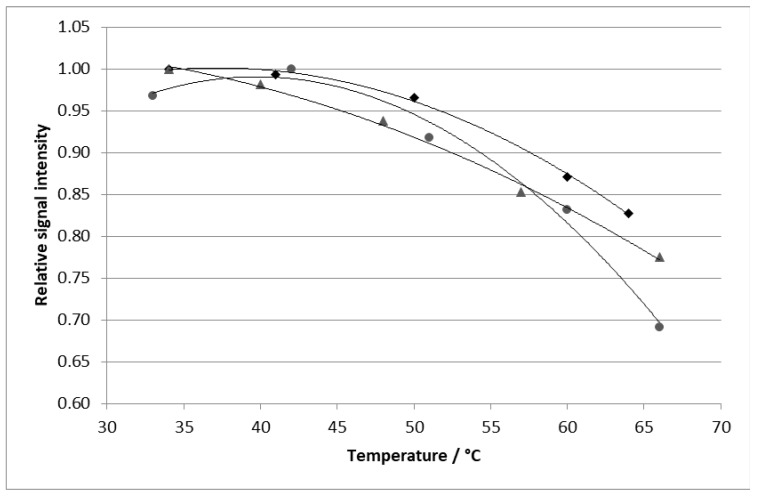
Relative luminescence intensity of chelate **1b** (square), **6b** (triangle) and **8b** (circle) vs. measurement temperature.

Finally, the dependency of luminescence on the measurement temperature between three chelates **1b**, **6b** and **8b** in Figure 3 shows increasing luminescence quenching in order **8b** > **6b** > **1b** being accordance with above observations, and is in agreement with the behavior of ILCT transition i.e., hypsochromic shift upon decreasing the temperature obtained earlier with phenylethynylpyri-dine Eu(III) chelates [29].

The luminescence intensities after drying are really remarkable e.g., when compared to the brightness of the well-known dissociation-enhanced lanthanide fluorescent immunoassays (DELFIA^®^) enhancement solution (26,320 M^−1 ^cm^−1^ in [30]) used in many sensitive bio-affinity assays. Therefore, it should be possible to design highly sensitive TR-FIA assays by using the novel labeling chelates. Actually, preliminary troponin I assays performed have given three, five and seven folds improved assay sensitivities by using e.g., chelate labels **3**, **6** and **8**, respectively.

## 3. Materials and Methods

### 3.1. Materials

All commercially available solvents and reagents were used without further purifications. MeCN, THF and TEA were dried with molecular sieves and K_2_CO_3_ was dried overnight at 140 °C before use. The organic intermediates were purified by silica gel 60 (Merck, Darmstadt, Germany) column chromatography. The low fluorescence MaxiSorp single wells made of irradiated polystyrene were purchased from Nunc (Thermo Fisher Scientific, Waltham, MA, USA). The monoclonal anti-troponin I detection antibodies were manufactured by International Point of Care Inc., Toronto, ON, Canada. The troponin I capture antibody and antigen were purchased from HyTest Ltd. (Turku, Finland).

### 3.2. Syntheses

The general synthetic pathways used for the Eu(III) Chelates **2**–**8** are shown in Scheme 2, Scheme 3, Scheme 4 and Scheme 5. The more detailed synthetic procedures and the results of the spectroscopic product characterization are presented in the Supporting information. Microwave assisted syntheses were performed with a Initiator+ microwave synthesizer from Biotage (Uppsala, Sweden).

The Eu(III) chelates were analysed and purified by using a reversed phase HPLC (2996 Photodiode Array Detector, 600 Controller, Delta 600 Fraction Collector III; Waters, Milford, MA, USA) with a RP-18 column. The solvents were A: triethylammonium acetate buffer (20 mM, pH 7) and B: 50% acetonitrile in triethylammonium acetate buffer (20 mM, pH 7). The gradient was started from 5% of solvent B and the amount of solvent B was linearly raised to 100% within 30 min.

The ^1^H and ^13^C-NMR spectra were recorded on an AVANCE 500 DRX (Bruker, Billerica, MA, USA). The chemical shifts are given in ppm from the internal tetramethylsilane. Mass spectra were recorded on Applied Biosystems QSTAR^®^ XL ESI-TOF (electrospray ionization-time-of-flight mass analyzer) instrument (Thermo Fisher Scientific) using α-cyano-4-cinnamic acid matrix. UV-Vis spectra were recorded on an Ultrospec^®^ 3300 pro (GE Healthcare Life Sciences, Chicago, IL, USA) or a UV-1800 spectrophotometer (Shimadzu, Kyoto, Japan).

*Eu(III) Chelate* (**1a**). A mixture of Eu(III) chelate **1** (2.5 μmol) and taurine (3.1 mg, 25 μmol) in aqueous 50 mM Na_2_CO_3_ buffer (0.125 mL, pH 9.8) was stirred overnight at RT (room temperature). The product was purified by HPLC. R_f_(HPLC): 15.1 min. UV: 320 nm.

*Triethyl 2,2′,2′′-[benzene-1,3,5-triyltris(oxy)]triacetate* (**9**). Ethyl bromoacetate (14.6 mL, 132 mmol) was added within 2 h to a mixture of benzene-1,3,5-triol (5.04 g, 40 mmol) and dry K_2_CO_3_ (18.2 g, 132 mmol) in dry MeCN (300 mL) at 50 °C under argon. After stirring for ca. 20 h at 50 °C, the mixture was filtered, the solid material washed with MeCN and the filtrate evaporated to dryness. The product was purified by silica gel column chromatography using MeOH/CH_2_Cl_2_ (first 0:100, then 1:99) as an eluent. Yield: 10.8 g (70%). ^1^H-NMR [DMSO-*d*_6_ (dimethyl sulfoxide), δ ppm]: 6.13 (3H, s), 4.74 (6H, s), 4.17 (6 H, q, *J* = 7.1 Hz), 1.22 (9H, t, *J* = 7.1 Hz). ^13^C-NMR (DMSO-*d*_6_, δ ppm): 169.02, 159.84, 95.12, 65.27, 61.10, 14.51. MS(ESI-TOF) calculated for C_18_H_24_O_9_ [M + H]^+^: 385,15, found: 385.04.

*Triethyl 2,2′,2′′-[(2-iodobenzene-1,3,5-triyl)tris(oxy)]triacetate* (**10**). The compound **9** (8.36 g, 22 mmol) in CH_3_COOH (40 mL) was added to a mixture of N-chlorosuccinimide (2.95 g, 22 mmol) and NaI (3.30 g, 22 mol) in CH_3_COOH (65 mL). After stirring for 1.5 h at RT, the mixture was evaporated to dryness. The residue was dissolved in EtOAc (ethyl acetate, 320 mL) and neutralized with saturated NaHCO_3_ solution. The phases were separated, the aqueous phase was extracted with EtOAc (80 mL) and the combined organic phases were dried with Na_2_SO_4_. Yield: 11,2 g (100%). ^1^H-NMR (CDCl_3_, δ ppm): 6.10 (2H, s), 4.65 (4H, s), 4.55 (4H, s), 4.27 (2H, q, *J* = 7.15 Hz), 4.26 (4H, q, *J* = 7.15 Hz), 1.31 (3H, t, *J* = 7.15 Hz), 1.30 (6H, t, *J* = 7.15 Hz). ^13^C-NMR (CDCl_3_, δ ppm): 168.14, 168.01, 159.89, 158.65, 94.34, 66.61, 65.61, 61.52, 61.43, 14.05. MS(ESI-TOF) calculated for C_18_H_23_IO_9_ [M + H]^+^: 511.05, found: 510.93.

*Diethyl 2,2′-[(4-bromo-1,3-phenylene)bis(oxy)]diacetate* (**11**). A mixture of 4-bromobenxene-1,3-diol (3.18 g, 16.8 mmol), dry K_2_CO_3_ (5,12 g, 37,0 mmol) and ethyl bromoacetate (4.11 mL, 37.0 mmol) in dry MeCN (60 mL) was stirred for 24 h at 50 °C. The mixture was filtered, the solid material washed with MeCN and the filtrate evaporated to dryness. The product was purified by silica gel column chromatography using MeOH/CH_2_Cl_2_ (first 0:100, then 1:99) as an eluent. Yield: 5.97 g (98%). ^1^H-NMR (DMSO-*d*_6_, δ ppm): 7.46, (1H, d, *J* = 8.8 Hz), 6.65 (1H, d, *J* = 2.7 Hz), 6.51 (1H, dd, *J* = 2.7 and 8.8 Hz), 4.91 (2H, s), 4.79 (2H, s), 4.18 (2H, q, *J* = 7.1 Hz), 4.16 (2H, q, *J* = 7.1 Hz), 1.22 (3 H, t, *J* = 7.1 Hz), 1.21 (3 H, t, *J* = 7.1 Hz). ^13^C-NMR (DMSO-*d*_6_, δ ppm): 168.65, 158.62, 155.08, 133.55, 108.65, 102.64, 102.09, 65.81, 65.44, 61.21, 61.15, 14.49. MS(ESI-TOF) calculated for C_14_H_17_BrO_6_ [M + H]^+^: 361.03 and 363.03 found: 360.89 and 362.92.

*General Procedure for the Synthesis of Compounds* **12** *and* **13**. A mixture of compound **10** or **11** (5.33 mmol), bis(triphenylphosphine)palladium(II) chloride (186 mg, 0.27 mmol), CuI (51 mg, 0.27 mmol), triphenylphosphine (142 mg, 0.54 mmol; only for compound **12**) in diethylamine (10 mL) and dry DMF (5 mL) was de-aerated with argon. After addition of trimethylsilylacetylene (1.17 mL, 8.34 mmol), the mixture was stirred for 45 or 30 min for compound **10** and **11**, respectively, at 100 °C using microwave heating. After evaporation to dryness, the residue was dissolved in CH_2_Cl_2_ (50 mL), washed with H_2_O (2 × 25 mL), dried with Na_2_SO_4_ and purified with column chromatography.

*Diethyl 2,2′-{{4-[(trimethylsilyl)ethynyl]-1,3-phenylene}bis(oxy)}diacetate* (**12**). The product was purified by silica gel column chromatography using EtOAc/petroleum ether (20:80) as an eluent. Yield: 94%. ^1^H-NMR (DMSO-*d*_6_, δ ppm): 7.31 (1H, d, *J* = 8.8 Hz), 6.52 (1 H, d, *J* = 2.4 Hz), 6.61 (1H, dd, *J* = 2.4 and 8.8 Hz), 4.85 (2H, s), 4.81 (2H, s), 4.18 (2H, q, *J* = 7.1 Hz), 4.17 (2H, q, *J* = 7.1 Hz), 1.23 (3H, t, *J* = 7.1 Hz), 1.21( 3H, t, *J* = 7.1 Hz), 0.22 (9H, s). ^13^C-NMR (DMSO-*d*_6_, δ ppm): 168.73, 160.08, 159.70, 134.94, 107.33, 105.18, 102.16, 100.66, 97.00, 65.66, 65.30, 61.17, 14.51, 14.49, 0.57. MS(ESI-TOF) calculated for C_19_H_22_O_6_Si [M + H]^+^: 379.16, found 379.39.

*Triethyl 2,2′,2′′-{{2-[(trimethylsilyl)ethynyl]benzene-1,3,5-triyl}tris(oxy)}triacetate* (**13**). The product was purified by silica gel column chromatography using EtOAc/petroleum ether (first 20:80, then 30:70) as an eluent. Yield: 70%. ^1^H-NMR (CDCl_3_, δ ppm): 6.10 (2H, s), 4.67 (4H, s), 4.55 (2H, s), 4.27 (2H, g, *J* = 7.15 Hz), 4.26 (4H, q, *J* = 7.15 Hz), 1.30 (6H, t, *J* = 7.15 Hz), 1.29 (3H, t, *J* = 7.15 Hz), 0.26 (9H, s). ^13^C-NMR (DMSO-*d*_6_, δ ppm): 168.60, 168.56, 160.98, 159.66, 101.34, 98.17, 95.29, 93.43, 65.77, 65.31, 61.06, 61.04, 14.41, 14.38, 0.56. MS(ESI-TOF) calculated for C_23_H_32_O_9_Si [M + H]^+^: 481.19, found: 481.99.

*General Procedure for the Synthesis of Compounds* **14** *and* **15**. A mixture of compound **12** or **13** (10.4 mmol) and tetrabutylammonium fluoride (3.25 g, 12.4 mmol) in CH_2_Cl_2_ (200 mL) stirred for 1.5 h at RT under argon. Mixture was washed with aqueous 10% citric acid solution (200 mL), H_2_O (200 mL), died with Na_2_SO_4_ and used for the next step without further purification as no impurities were found with the TLC analysis.

*Diethyl 2,2′-{[4-(ethynyl)-1,3-phenylene]bis(oxy)}diacetate* (**14**). Yield: 100%. ^1^H-NMR (DMSO-*d*_6_, δ ppm): 7.34 (1H, d, *J* = 8.8 Hz), 6.53 (1H, d, *J* = 2.4 Hz), 6.52 (1H, dd, *J* = 2.4 and 8.8 Hz), 4.89 (2H, s), 4.81 (2H, s), 4.17 (2H, q, *J* = 7.1 Hz), 4.16 (2H, q, *J* = 7.1 Hz), 4.10 (1H, s), 1.22 (3H, t, *J* = 7.1 Hz), 1.21 (3H, t, *J* = 7.1 Hz). ^13^C-NMR (DMSO-*d*_6_, δ ppm): 168.84, 168.83, 160.29, 159.59, 134.90, 107.29, 104.62, 100.49, 83.70, 80.35, 65.40, 65.29, 61.17, 14.49. MS(ESI-TOF) calculated for C_16_H_18_O_6_ [M + H]^+^: 307.12, found: 307.01.

*Triethyl 2,2′,2′′-{[2-(ethynyl]benzene-1,3,5-triyl]tris(oxy)}triacetate* (**15**). Yield: 100%. ^1^H-NMR (CDCl_3_, δ ppm): 6.06 (2H, s), 4.69 (4H, s), 4.55 (2H, s), 4.27 (2H, q, *J* = 7.15 Hz), 4.26 (4H, q, *J* = 7.15 Hz), 3.50 (1H, s), 1.30 (3H, t, *J* = 7.15 Hz), 1.29 (6H, t, *J* = 7.15 Hz). ^13^C-NMR (CDCl_3_, δ ppm): 168.23, 168.10, 161.48, 159.39, 95.25, 94.18, 85.18, 66.42, 65.49, 61.64, 61.49, 14.14. MS(ESI-TOF) calculated for C_20_H_24_O_9_ [M + H]^+^: 409.15, found: 409.20.

*General Procedure for the Synthesis of Ligand Esters* **17** and **18**. A mixture of compound **16** (0.22 g, 0.25 mmol; [31]) and compound **14** or **15** (0.60 mmol) in dry TEA (2 mL) and THF (4 mL) was de-aerated with argon. After an addition of bis(triphenylphosphine)palladium(II) dichloride (11 mg, 16 μmol) and CuI (6 mg, 32 μmol), the mixture was stirred overnight at 55 °C and evaporated to dryness. The residue was dissolved in CH_2_Cl_2_ (30 mL), washed with H_2_O (2 × 15 mL), dried with Na_2_SO_4_ and purified by column chromatography.

*Ligand Ester* **17**. The product was purified by silica gel column chromatography using TEA/EtOAc/petroleum ether (20:40:40) as an eluent. Yield: 48%. ^1^H-NMR (DMSO-*d*_6_, δ ppm): 7.47 (2 H, d, *J* = 1.1 Hz), 7.45 (2H, d, *J* = 8.6 Hz), 7.43 (2H, d, *J* = 1.1 Hz), 6.79 (2H, d, *J* = 8.4 Hz), 6.61 (2H, d, *J* = 2.3 Hz), 6.55 (2H, dd, *J* = 8.4 and 2.3 Hz), 6.44 (2H, d, *J* = 8.4 Hz), 4.91 (4H, s), 4.83 (4H, s), 4.75 (2H, s), 4.17 (4H, q, *J* = 7.1 Hz), 4.17 (4H, q, *J* = 7.1 Hz), 4.05 (4H, q, *J* = 7.1 Hz), 3.94 (4H, s), 3.82 (4H, s), 3.56 (8H, s), 2.65 (4H, s), 1.23 (6H, t, *J* = 7.1 Hz), 1.20 (6H, t, *J* = 7.1 Hz), 1.16 (12H, t, *J* = 7.1 Hz). ^13^C-NMR (DMSO-*d*_6_, δ ppm): 171.14, 168.77, 168.702, 160.28, 160.14, 159.58, 159.21, 146.96, 134.86, 129.34, 129.28, 129.18, 127.50, 122.84, 122.50, 114.42, 107.61, 104.26, 100.64, 90.62, 90.38, 65.65, 65.35, 61.21, 61.19, 59.73, 59.60, 56.67, 54.98, 32.61, 14.50, 14.49, 14.46. MS(ESI-TOF) calculated for C_70_H_84_N_6_O_20_ [M + 2H]^+^: 1329.59, found: 1330.66.

*Ligand Ester* **18**. The product purified by silica gel column chromatography using MeOH/CH_2_Cl_2_ (5:95) as an eluent. Yield: 73%. ^1^H-NMR (CDCl_3_, δ ppm): 7.56 (2H, s), 7.51 (2H, s), 6.88 (2H, d, *J* = 8.3 Hz), 6.56 (2H, d, *J* = 8.3 Hz), 5.98 (4H, s), 4.70 (8H, s), 4.52 (4H, s), 4.28 (4H, q, *J* = 7.1 Hz), 4.26 (8H, q, *J* = 7.1 Hz), 4.15 (8H, q, *J* = 7.1 Hz), 4.04 (4H, s), 3.87 (4H, s), 3.62 (8H, s), 2.80–2.70 (4H, m), 1.65 (2H, bs), 1.31 (6H, t, *J* = 7.1 Hz), 1.25 (12H, t, *J* = 7.1 Hz), 1.24 (12H, t. *J* = 7.1 Hz). ^13^C-NMR (CDCl_3_, δ ppm): 171.14, 168.45, 168.14, 160.91, 159.58, 159.52, 157.81, 144.59, 133.17, 130.33, 129.52, 123.52, 122.58, 115.26, 96.52, 95.52, 93.46, 85.52, 66.04, 65.29, 61.54, 61.32, 60.49, 60.35, 59.92, 56.02, 54.88, 32.58, 14.24, 14.16, 14.15. MS(ESI-TOF) calculated for C_78_H_96_N_6_O_26_ [M + 2H]^+^: 1533.65, found: 1532.96.

*General Procedure for the Synthesis of Eu(III) Chelates* **19** *and* **20**. Compound **17** or **18** (52 μmol) in 0.5 M KOH in EtOH (5.4 mL) was stirred for 30 min at RT, H_2_O (2.7 mL) was added and the mixture was further stirred at RT for 2 h. After evaporation of EtOH and an additional stirring for 2 h at RT, the pH was adjusted to 6.5 by addition of 6 M HCl. EuCl_3_ (21 mg, 57 μmol) in water (0.5 mL) was added within 5 min and the pH was maintained at 6.0–6.5 with suitable additions of solid NaHCO_3_. After stirring overnight at RT, the pH was adjusted to 8.5 with 1 M NaOH. The precipitate was removed by centrifugation and the supernatant evaporated to dryness. The product was purified by HPLC.

*Eu(III) Chelate* **19**. Yield: 88%. R_f_(HPLC): 14.8 min. UV: 342 nm.

*Eu(III) Chelate* **20**. Yield: 92%. R_f_(HPLC): 12.3 min. UV: 346 nm.

*General Procedure for the Synthesis of Eu(III) Chelates* **2** *and* **3**. An aqueous solution (1.3 mL) of the Eu(III) chelate **19** or **20** (55 μmol) was added within 5 min to a mixture of CSCl_2_ (30 µL, 0.39 mmol) and NaHCO_3_ (37 mg, 0.44 mmol) in CHCl_3_ (1.3 mL). After stirring for 45 min at RT, the two phases were separated and the aqueous phase was washed with CHCl_3_ (3 × 1.3 mL). The product was precipitated with acetone (ca. 45 mL), isolated by centrifugation, washed with acetone (2 × 10 mL) and dried overnight in vacuum desiccator. The products were used as such for next phase or for labeling the antibodies.

*Eu(III) Chelate* **2**. R_f_(HPLC): 18.4 min. UV: 346 nm. 

*Eu(III) Chelate* **3**. R_f_(HPLC): 14.6 min. UV: 350 nm. 

*General Procedure for the Synthesis of Eu(III) Chelates* **2a** *and* **3a**. A mixture of Eu(III) chelate **2** or **3** (2.5 μmol) and taurine (3.1 mg, 25 μmol) was stirred in aqueous 50 mM Na_2_CO_3_ buffer (0.125 mL, pH 9.8) for overnight at RT. The product was purified by HPLC.

*Eu(III) Chelate* **2a**. R_f_(HPLC): 15.1 min. UV: 340 nm.

*Eu(III) Chelate* **3a**. R_f_(HPLC): 12.5min. UV: 351 nm.

*N-(4-Ethynylphenyl)-2,2,2-trifluoroacetamide* (**21**). 4-Ethynylaniline (1.17 g, 10 mmol) was added in small portions to a ice-cold trifluoroacetic anhydride (5.6 mL, 40 mmol). After stirring for 2.5 h at RT, cold H_2_O was added, the mixture filtrated and the product washed with cold H_2_O. Yield: 2.12 g (98%). ^1^H-NMR (CDCl_3_, δ ppm): 8.08 (1H, bs), 7.55 (2H, d, *J* = 8.8 Hz), 7.51 (2 H, d, *J* = 8.8 Hz), 3.1 (1H, s). ^13^C-NMR (CDCl_3_, δ ppm): 154.72, 135.40, 133.23, 129.92, 120.25, 120.01, 82.67, 77.99. MS(ESI-TOF) calculated for C_10_H_6_F_3_NO [M + H]^+^: 213.04, found 213.05.

*N-{4-{[2,6-Bis(hydroxymethyl)pyridin-4-yl]ethynyl}phenyl}-2,2,2-trifluoroacetamide* (**23**) A mixture of the compound **21** (0.55 g, 2.58 mmol) and 4-bromo-2,6-dihydroxymethylpyridine (**22**; [28]) (0.47 g, 2.15 mmol) in dry TEA (5 mL) and THF (10 mL) was de-aerated with argon. After addition of bis(triphenylphosphine)palladium(II) chloride (30 mg, 43 μmol) and CuI (16 mg, 86 μmol), the mixture was stirred for 19 h at 55 °C. After evaporation to dryness, the residue was treated with a cold mixture of CH_2_Cl_2_ (40 mL) and H_2_O (20 mL), filtered and the product washed with cold H_2_O (10 mL) and CH_2_Cl_2_ (10 mL). Yield: 0.56 g (75%). ^1^H-NMR (DMSO-*d*_6_, δ ppm): 11.5 (1H, bs), 7.79 (2H, d, *J* = 8.8 Hz), 7.68 (2H, d, *J* = 8.8 Hz), 7.41 (2H, s), 5.49 (2H, bs), 4.56 (4H, s). ^13^C-NMR (DMSO-*d*_6_, δ ppm): 162.25, 155.53, 155.23, 154.94, 154.64. 138.01, 133.12, 131.39, 121.52, 120.00, 119.58, 118.63, 117.29, 114.99, 92.93, 87.98, 64.39. MS(ESI-TOF) calculated for C_17_H_13_F_3_N_2_O_3_ [M + H]^+^: calculated 351.10, found 351.96.

*N-{4-{[2,6-bis(bromomethyl)pyridin-4-yl]ethynyl}phenyl}-2,2,2-trifluoroacetamide* (**24**). PBr_3_ (0.35 mL, 3.67 mmol) was added in a suspension of the compound **23** (0.86 g, 2.45 mmol) in CHCl_3_ (100 mL). After stirring for 20 h at 60 °C, the mixture was neutralized with 5% NaHCO_3_ (50 mL). The aqueous phase was extracted with CHCl_3_ (50 mL) and the combined organic phases were dried with Na_2_SO_4_, filtered and evaporated to dryness. The product **24** (1.09 g, 93%) was used for the next step without further purifications. ^1^H-NMR (CDCl_3_, δ ppm): 7.93 (1H, s), 7.63 (2H, d, *J* = 8.7 Hz), 7.59 (2H, d, *J* = 8.7 Hz), 7.47 (2H, s), 4.53 (4H, s). ^13^C-NMR (CDCl_3_, δ ppm): 157.02, 155.16, 154.85, 154.55, 154.23, 134.92, 133.36, 133.11, 124.55, 120.26, 119.73, 118.98, 116.68, 114.38, 112.10, 93.70, 86.77, 32.98. MS(ESI-TOF) calculated for C_17_H_11_Br_2_F_3_N_2_O [M + H]^+^: 474.93, 476.93, 478.92, found 475.38, 477.44, 479.45.

*Ethyl-2-{[4-bromo-6-(carboxyethyl)pyridine-2-yl]methylenenitrilo}acetate* (**26**). A mixture of 4-bromo-6-bromomethyl-2-carboxyethylpyridine (**25**; [14]) (1.66 g, 5.15 mmol), glycine ethyl ester hydrochloride (3.60 g, 25.8 mmol) and di-isopropylethylamine (9.1 mL) in dry MeCN (70 mL) was stirred overnight at RT. The mixture was evaporated to dryness and the product was purified by silica gel column chromatography using MeOH/CH_2_Cl_2_ (3:97) as an eluent. Yield 1.55 g (87%). ^1^HNMR: (CDCl_3_, δ ppm): 8.14 (1H, d, *J* = 1.65 Hz), 7.85 (1H, d, *J* = 1.60 Hz), 4.47 (2H, q, *J* = 7.12 Hz), 4.20 (2H, q, *J* = 7.13 Hz), 4.05 (2H, s), 3.47 (2H, s), 2.26 (1H, s), 1.43 (3H, t, *J* = 7.13 Hz), 1.28 (3H, t, *J* = 7.13 Hz). ^13^C-NMR (DMSO-*d*_6_, δ ppm): 172.41, 164.08, 162.23, 148.76, 133.81, 128.53, 126.29, 62.07, 60.47, 53.65, 50.24, 14.59, 14.54. MS(ESI-TOF) calculated for C_13_H_17_BrN_2_O_4_ [M + H]^+^: 345.05, 347.05, found 344.97, 346.97.

*Synthesis of Compound* **27**. A mixture of compounds **24** (0.44 g, 0.94 mmol), **26** (0.65 g, 1.89 mmol), dry K_2_CO_3_ (0.52 g, 3.76 mmol) was stirred in dry MeCN (30 mL) for 27 h at 70 °C. The mixture was filtrated and the filtrate evaporated to dryness, and the product was purified by silica gel column chromatography using TEA/EtOAc/petroleum ether (1:69:30) as an eluent. Yield: 66%. ^1^H-NMR (CDCl_3_, δ ppm): 8.53 (1H, s), 8.14 (2H, d, *J* = 1.80 Hz), 8.11 (2H, d, *J* = 1.80 Hz), 7.68 (2 H, d, *J* = 8.73 Hz), 7.60 (2H, d, *J* = 8.73 Hz), 7.34 (2H, s), 4.44 (4H, q, *J* = 7.08 Hz), 4.18 (4 H, q, *J* = 7.15 Hz); 4.10 (4H, s), 3.98 (4 H, s), 3.50 (4 H, s), 1.40 (6H, t, *J* = 7.08 Hz), 1.28 (6H, t, *J* = 7.15 Hz). ^13^C-NMR (CDCl_3_, δ ppm): 171.02, 164.11, 161.85, 158.48, 155.23, 154.92, 154.62, 154.32, 148.52, 136.02, 134.37, 132.98, 132.16, 129.28, 128.35, 127.01, 123.43, 120.28, 119.04, 116.74, 114.45, 112.15, 92.79, 87.68, 62.23, 60.68, 59.82, 59.64, 55.41, 14.24. MS(ESI-TOF) calculated for C_43_H_43_Br_2_F_3_N_6_O_9_ [M + H]^+^: 1003.15, 1005.15, 1007.15, found 1003.71, 1005.48, 1007.58.

*General Procedure for the Synthesis of Ligand Esters* **28** *and* **29**. A mixture of compound **27** (0.120 g, 0.148 mmol), compound **14** or **15** (0.355 mmol) in dry TEA (1 mL) and THF (2 mL) was de-aerated with argon. After an addition of bis(triphenylphosphine)palladium(II) dichloride (10 mg, 14 μmol) and CuI (6 mg, 28 μmol), the mixture was stirred overnight at 55 °C and evaporated to dryness. The residue was dissolved in CH_2_Cl_2_ (30 mL), washed with H_2_O (2 × 15 mL), dried with Na_2_SO_4_ and purified by silica gel column chromatography.

*Ligand Ester* **28**. The product purified by silica gel column chromatography using 10% EtOH/CH_2_Cl_2_ as an eluent. Yield: 69%. ^1^H-NMR (DMSO-*d*_6_, δ ppm): 11.44 (1H, s), 7.85 (2H, s), 7.77 (2H, s), 7.69 (2H, d, *J* = 8.1 Hz), 7.57 (2H, d, *J* = 6.7 Hz), 7.45 (2H, d, *J* = 8.1 Hz), 7.43 (2H, s), 6.60 (2H, s), 6.53 (2H, d, *J* = 6.7 Hz), 4.91 (4H, s), 4.82 (4 H, s), 4.30 (4H, q, *J* = 6.7 Hz), 4.17 (8H, q, *J* = 7.1 Hz), 4.06 (4H, q, *J* = 6.9 Hz), 4.00 (4 H, s), 3.95 (4 H, s), 3.53 (4 H, s), 1.30 (6H, t, *J* = 6.7 Hz), 1.22 (6H, t. *J* = 7.1 Hz), 1.20 (6H, t, *J* = 7.1 Hz), 1.18 (6H, t, *J* = 6.9 Hz). ^13^C-NMR (DMSO-*d*_6_, δ ppm): 171.19, 168.67, 168.62, 164.58, 160.62, 160.32, 159.04, 155.47, 155.18, 154.89, 154.59, 147.74, 137.93, 134.95, 133.48, 132.98, 130.67, 129.50, 127.50, 124.78, 123.44, 121.34, 119.46, 118.50, 117.26, 114.96, 112.67, 107.69, 103.73, 100.64, 92.13, 89.65, 87.58, 65.70, 65.34, 61.84, 61.20, 60.48, 59.64, 56.50, 55.71, 55.38, 14.51, 14.46. MS(ESI-TOF) calculated for C_75_H_77_F_3_N_6_O_21_ [M + H]^+^: 1455.52, found 1455.36.

*Ligand Ester* **29**. The product was purified by silica gel column chromatography using TEA/EtOAc/petroleum ether (1:69:30) as an eluent. Yield: 57%. ^1^H-NMR (CDCl_3_, δ ppm): 9.12 (1H, s), 8.09 (2H, d, *J* = 1.25 Hz), 8.06 (2H, d, *J* = 1.25 Hz), 7.57 (2 H, s), 7.34 (2H, d, *J* = 8.80 Hz), 7.30 (2H, d, *J* = 8.80 Hz), 5.97 (4H, s), 4.63 (8H, s), 4.60 (4H, s), 4.45 (4H, q, *J* = 7.12 Hz), 4.30 (4H, q, *J* = 7.12 Hz), 4,21 (8H, q, *J* = 7.12 Hz), 4.17 (4H, q, *J* = 7.12), 4.10 (4H, s), 4.02 (4H, s), 3.52 (4H, s), 1.42 (6H, t, *J* = 7.12 Hz), 1.33 (6H, t, *J* = 7.12 Hz), 1.29 (6H, *J* = 7.12 Hz), 1.25 (12H, t, *J* = 7.12 Hz). ^13^C-NMR (CDCl_3_, δ ppm): 171.13, 168.69, 168.69, 165.07, 161.08, 160.53, 159.98, 159.03, 154.95, 154.66, 147.65, 136.35, 133.99, 132.56, 132.17, 128.47, 127.64, 125.43, 123.12, 119.95, 116.90, 114.59, 96.39, 94.76, 93.71, 92.65, 87.75, 87.21, 66.12, 65.22, 61.91, 61.89, 61.51, 60.57, 60.10, 59.94, 55.11, 14.32, 14.27, 14.13. MS(ESI-TOF) calculated for C_83_H_89_F_3_N_6_O_27_ [M + H]^+^: 1659.58, found 1659.81.

*General Procedure for the Synthesis of Eu(III) Chelates* **30** *and* **31**. Compound **28** or **29** (90 μmol) was stirred in 0.5 M KOH in EtOH (13 mL) for 30 min at RT. H_2_O (10 mL) was added and the mixture was further stirred at RT for 3 h. After evaporation of EtOH and an additional overnight stirring at RT, the pH was adjusted to 6.5 by addition of 6 M HCl. EuCl_3_ (33 mg, 90 μmol) in water (0.5 mL) was added within 5 min and the pH was maintained at 6.0–6.5 with suitable additions of solid NaHCO_3_. After stirring overnight at RT, the pH was adjusted to 8.5 with 1 M NaOH. The precipitate was removed by centrifugation and the supernatant evaporated to dryness. The product was purified by HPLC.

*Eu(III) Chelate* **30**. Yield: 100%. R_f_(HPLC): 14.2 min. UV: 340 nm.

*Eu(III) Chelate* **31**. Yield: 100%. R_f_(HPLC): 14.5 min. UV: 359 nm.

*General Procedure for the Synthesis of Eu(III) Chelates* **4** *and* **5**. An aqueous solution (2.7 mL) of the Eu(III) chelate **30** or **31** (0.10 mmol) was added within 5 min to a mixture of CSCl_2_ (53 µL, 0.70 mmol) and NaHCO_3_ (67 mg, 0.80 mmol) in CHCl_3_ (2.7 mL). After stirring for 30 min at RT, the two phases were separated and the aqueous phase was washed with CHCl_3_ (3 × 3 mL). The product was precipitated with acetone (ca. 45 mL), isolated by centrifugation, washed with acetone (2 × 10 mL) and dried overnight in vacuum desiccator.

*Eu(III) Chelate* **4**. R_f_(HPLC): 18.4 min. UV: 346 nm.

*Eu(III) Chelate* **5**. R_f_(HPLC): 20.9 min. UV: 325 (sh), 340 and 362 (sh) nm.

*General Procedure for the Synthesis of Eu(III) Chelates* **4a** *and* **5a**. A mixture of Eu(III) chelate **4** or **5** (6.3 μmol) and taurine (8 mg, 63 μmol) was stirred in aqueous 50 mM Na_2_CO_3_ buffer (0.64 mL, pH 9.8) and DMF (0.64 mL) for overnight at RT. The product was purified by HPLC.

*Eu(III) Chelate* **4a**. R_f_(HPLC): 16.7 min. UV: 340 nm.

*Eu(III) Chelate* **5a**. R_f_(HPLC): 14.3 min. UV: 349 nm.

*Diethyl 2,2′-{[(4-bromopyridine-2,6-diyl)bis(methylene)]bis(azanediyl)}diacetate* (**33**). A mixture of 4-bromo-2,6-dibromomethylpyridine (**32**, [28]) (3.50 g, 10 mmol), glycine ethyl ester hydrochloride (14.0 g, 0.10 mol) and di-isopropylethylamine (35 mL) in dry MeCN (130 mL) was stirred overnight at RT. After evaporation to dryness, the residue was dissolved in CH_2_Cl_2_ (100 mL), washed with H_2_O (3 × 50 mL), dried with Na_2_SO_4_, and the product was purified by silica gel column chromatography using TEA/EtOAc/petroleum ether (first 10:20:70, then 15:30:55) as an eluent. Yield 2.48 g (64%). ^1^H-NMR (CDCl_3_, δ ppm): 7.43 (2H, s), 4.20 (4H, q, *J* = 7.2 Hz), 3.91 (4H, s), 3.46 (4H, s), 2.25–2.15 (2H, bs), 1.28 (6H, t, *J* = 7.2 Hz). ^13^C-NMR (CDCl3, δ ppm): 172.00, 160.40, 133.83, 60.75, 54.00, 50.34, 14.18. MS(ESI-TOF) calculated for C_15_H_22_BrN_3_O_4_ [M + H]^+^: 388.09 and 390.09, found 388.75 and 390.75.

*Ethyl 4-bromo-6-{{{{4-bromo-6-{[(2-ethoxy-2-oxoethyl)amino]methyl}pyridin-2-yl}methyl}(2-ethoxy-2-oxo-ethyl)amino}methyl}picolinate* (**34**). 4-Bromo-6-bromomethyl-2-carboxyethylpyridine (**25**, [14]) (2.06 g, 6.39 mmol) was added in small portions to a mixture of the compound **33** (2.49 h, 6.39 mmol), dry K_2_CO_3_ (3.53 g, 25.6 mmol) in dry MeCN (215 mL) within 2 h at RT. After stirring for 22 h at RT, the mixture was filtrated and the filtrate evaporated to dryness. The product was purified by silica gel column chromatography using TEA/EtOAc/petroleum ether (first 10:25:65) as an eluent. Yield 2.03 g (50%). ^1^H-NMR (CDCl_3_, δ ppm): 8.12 (1H, d, *J* = 1.7 Hz), 8.07 (1H, d, *J* = 1.7 Hz), 7.55 (1H, d, *J* = 1.5 Hz), 7.43 (1H, d, *J* = 1.5 Hz), 4.46 (2H. q, *J* = 7.2 Hz), 4.19 (4H, q, *J* = 7.2 Hz), 4.09 (2H, s), 3.95 (2H, s), 3.90 (2H, s), 2.3–2.1 (1H, bs), 1.43 (3H, t, *J* = 7.2 Hz), 1.29 (3H, t, *J* = 7.2 Hz), 1.29 (3H, t, *J* = 7.2 Hz). ^13^C-NMR (CDCl3, δ ppm): 171.98, 170.81, 164.03, 161.57, 160.41, 159.70, 148.54, 134.26, 133.90, 129.13, 126.95, 124.60, 123.77, 62.16, 60.75, 60.63, 59.61, 59.48, 55.38, 54.00, 50.36, 14.19, 14.17, 14.14. MS(ESI-TOF) calculated for C_24_H_30_Br_2_N_4_O_6_ [M + H]^+^: 629.06, 631.05 and 633.06, found 629.54, 631.56 and 633.54.

*Ethyl 6-(hydroxymethyl)-4-{[4-(2,2,2-trifluoroacetamido)phenyl]ethynyl}picolinate* (**36**). A mixture of the compound **21** (0.34 g, 1.60 mmol) and **35** (0.47 g, 2.15 mmol; [14]) in dry TEA (5 mL) and THF (10 mL) was de-aerated with argon. After addition of bis(triphenylphosphine)palladium(II) chloride (19 mg, 27 μmol) and CuI (10 mg, 53 μmol), the mixture was stirred for 24 h at 55 °C. After evaporation to dryness, the product was purified by silica gel column chromatography using EtOH/CH_2_Cl_2_/TEA (10:89:1) as an eluent. Yield: 0.49 g (94%). ^1^H-NMR (CDCl_3_, δ ppm): 8.49 (1H, s), 8.08 (1H, s), 7.66 (2H, d, *J* = 8.7 Hz), 7.60 (1H, s), 7.59 (2H, d, *J* = 8.7 Hz), 4.85 (2H, s), 4.49 (2 H, q, *J* = 7.1 Hz), 1.43 (3H, t, *J* = 7.1 Hz). ^13^C-NMR (CDCl_3_, δ ppm): 164.55, 160.42, 155.24, 154.94, 154.64, 154.34, 147.42, 136.24, 133.10, 132.99, 125.58, 125.27, 120.30, 119.36, 118.94, 116.65, 114.35, 112.06, 94.24, 87.57, 64.30, 62.10, 14.18. MS(ESI-TOF) calculated for C_19_H_15_F_3_N_2_O_4_ [M + H]^+^ 393.18; found 394.16.

*Ethyl 6-(bromomethyl)-4-{[4-(2,2,2-trifluoroacetamido)phenyl]ethynyl}picolinate* (**37**). A mixture of compound **36** (2.10 g, 5.35 mmol) and PBr_3_ (0.75 mL, 8.03 mmol) in dry CHCl_3_ (180 mL) was stirred for 18 h at 55 °C, neutralized with aqueous 5% NaHCO_3_ (150 mL), the aqueous phase was extracted with CHCl_3_ (2 × 50 mL) and the combined organic phases were dried with Na_2_SO_4_. The product was purified by silica gel column chromatography using EtOH/CH_2_Cl_2_ (10:90) as an eluent. Yield: 2.01 g (84%). ^1^H-NMR (CDCl_3_, δ ppm): 8.25 (1H, s), 8.01 (1H, d, *J* = 1.1 Hz), 7.75 (1H, d, *J* = 1.1 Hz); 7.68 (2H, d, *J* = 8.7 Hz), 7.65 (2H, d, *J* = 8.7 Hz); 4.62 (2H, s), 4.50 (2H, q, *J* = 7.1 Hz), 1.45 (3H, t, *J* = 7.1 Hz). ^13^C-NMR (CDCl_3_, δ ppm): 164.32, 157.68, 155.16, 154.85, 154.55, 154.25, 148.06, 136.21, 133.59, 133.06, 128.36, 126.09, 120.26, 119.32, 118.92, 116.62, 114.32, 112.03, 94.61, 86.29, 62.25, 32.62, 14.20. MS(ESI-TOF) calculated for C_19_H_14_BrF_3_N_2_O_3_ [M + H]^+^ 455.02 and 457.02; found 455.78 and 457.73.

*Compound* **38**. A mixture of compound **34** (2.02 g, 3.20 mmol), compound **37** (1.46 g, 3.20 mmol), dry K_2_CO_3_ (1.77 g, 12.8 mmol) in dry MeCN (70 mL) was stirred for 18 h at RT under argon. The mixture was filtrated and the filtrate evaporated to dryness. The product was purified by silica gel column chromatography using EtOH/CH_2_Cl_2_ (first 5:95, then 10:90) as an eluent. Yield: 3.03 g (94%). ^1^H-NMR (DMSO-*d*_6_, δ ppm): 11.46 (1H, s), 7.97, (1H, s), 7.90 (1H, s), 7.82–7.77 (4H, m), 7.65 (2H, d, *J* = 8.4 Hz), 7.58 (1H, s), 7.54 (1H, s), 4.35 (2H, q, *J* = 7.1 Hz), 4.32 (2H, q, *J* = 7.2 Hz), 4.07 (2H, q, *J* = 7.1 Hz), 4.06 (2H, q, *J* = 7.1 Hz), 4.01 (2H, s), 3.98 (2H, s), 3.90 (2 H, s), 3.89 (2H, s), 3.57 (2H, s), 3.53 (2H, s), 1.34 (3H, t, H = 7.1Hz),1.31 (3H, t, *J* = 7.2 Hz), 1.20 (3H, t, *J* = 7.1 Hz), 1.18 (3H, t, *J* = 7.1 Hz). ^13^C-NMR (DMSO-*d*_6_, δ ppm): 170.71, 170.69, 170.68, 170.56, 164.00, 163.38, 161.62, 160.41, 159.98, 159.81, 154.94, 154.64, 154.36, 154.03, 148.04, 147.33, 137.57, 135.94, 133.09, 132.78, 132.66, 132.62, 131.45, 128.42, 125.84, 120.81, 120.61, 118.07, 116.68, 114.39, 112.06, 93.81, 86.24, 61.43, 61.28, 61.14, 58.89, 58.19, 58,13, 58.94, 58.88, 55.32, 55.21, 14.07, 14.01, 13.96, 13.94. MS(ESI-TOF) calculated for C_43_H_43_Br_2_F_3_N_6_O_9_ [M + H]^+^ 1003.22, 1005.22 and 1007.22; found 1003.59, 1005.48 and 1007.40.

*Ligand Ester* **39**. A mixture of compound **38** (0.23 g, 0.23 mmol), compound **15** (0.22 g, 0.55 mmol) in dry TEA (2 mL) and THF (4 mL) was de-aerated with argon. After an addition of bis(triphenyl-phosphine)palladium(II) dichloride (10 mg, 14 μmol) and CuI (6 mg, 28 μmol), the mixture was stirred overnight at 55 °C and evaporated to dryness. The product was purified by silica gel column chromatography using EtOH/CH_2_Cl_2_/TEA (10:89/1) as an eluent. Yield: 0.31 g (57%). ^1^H-NMR (DMSO-*d*_6_, δ ppm): 11.43 (1H, s), 7.90 (1H, s), 7.87 (1H, s), 7.83 (1H, s), 7.77 (1H, s), 7.71 (2H, d, *J* = 8.6 Hz), 7.60 (2H, d, *J* = 8.6 Hz), 7.38 (1H, s), 7.37 (1H, s), 4.87 (4H, s), 4.83 (4H, s), 4.82 (2H, s), 4.79 (2H, s), 4.32 (2H, q, *J* = 7.1 Hz), 4.31 (2H, q, *J* = 7.1 Hz), 4.18 (4H, q, *J* = 7.1 Hz), 4.17 (2H, s), 4.16 (4H, q, *J* = 7.1 Hz), 4.13 (4H, q, *J* = 7.1 Hz), 4.06 (2H, q, *J* = 7.2 Hz), 4.05 (2H, q, *J* = 7.2 Hz), 4.04 (2H, s), 3.96 (2H, s), 3.95 (2H, s), 3.54 (2H, s), 3.52 (2H, s), 1.31 (3H, t, *J* = 7.1 Hz), 1.30 (3H, t, *J* = 7.1 Hz), 1.22 (6H, t, *J* = 7.1 Hz), 1.20 (6H, t, *J* = 7.1 Hz), 1.18 (6H, t, *J* = 7.1 Hz), 1.17 (3H, t, *J* = 7.2 Hz), 1.16 (3H, t, *J* = 7.2 Hz). ^13^C-NMR (DMSO-*d*_6_, δ ppm): 171.27, 171.21, 168.60, 164.73, 164.62, 162.78, 161.07, 160.92, 160.66, 160.55, 158.80, 158.68, 155.53, 155.25, 154.96, 154.65, 147.81, 138.43, 133.45, 133.10, 132.74, 132.13, 127.72, 127.21, 124.93, 124.63, 123.14, 123.08, 121.35, 119.62, 118.07, 117.33, 115.04, 112.74, 94.65, 94.43, 94.29, 93.93, 93.88, 93.44, 93.42, 89.09, 87.53, 86,80, 65.83, 65.76, 65.46, 65.44, 61.82, 61.75, 61.19, 61.17, 61.14, 60.40, 60.35, 59.59, 59.53, 59.29, 59.25, 55.37, 55.02, 14.50, 14.48, 14.47, 14.46, 14.45, 14.44, 14.42. MS(ESI-TOF) calculated for C_83_H_89_F_3_N_6_O_27_ [M + H]^+^ 1659.58; found 1659.34.

*Eu(III) Chelates* **40**. The compound **39** (0.28 g, 0.169 mmol) in 0.5 M KOH in EtOH (18.2 mL) was stirred for 30 min at RT, H_2_O (10 mL) was added and the mixture was further stirred at RT for 7 h. After evaporation of EtOH and an additional overnight stirring at RT, the pH was adjusted to 6.5 by addition of 6 M HCl. EuCl_3_ (68 mg, 0.186 μmol) in water (1.0 mL) was added within 5 min and the pH was maintained at 6.0–6.5 with suitable additions of solid NaHCO_3_. After stirring overnight at RT, the pH was adjusted to 8.5 with 1 M NaOH. The precipitate was removed by centrifugation and the supernatant evaporated to dryness. The product was purified by HPLC. Yield: 0.20 g (56%). R_f_(HPLC): 15.2 min. UV: 358 nm.

*Eu(III) Chelate* **6**. An aqueous solution (2.25 mL) of the Eu(III) chelate **40** (0.13 g, 61 μmol) was added within 5 min to a mixture of CSCl_2_ (64 µL, 0.85 mmol) and NaHCO_3_ (82 mg, 0.97 mmol) in CHCl_3_ (2.25 mL). After stirring for 15 min at RT, the two phases were separated and the aqueous phase was washed with CHCl_3_ (3 × 2.25 mL). The product was precipitated with acetone (ca. 45 mL), isolated by centrifugation, washed with acetone (2 × 10 mL) and dried overnight in vacuum desiccator over silica gel. Yield: 0.12 g. R_f_(HPLC): 21.9 min. UV: 342 nm.

*Eu(III) Chelate* **6a**. A mixture of Eu(III) chelate **6** (25 mg 11.7 μmol) and taurine (15 mg, 0.117 mmol) in aqueous 50 mM Na_2_CO_3_ buffer (0.88 mL, pH 9.8) was stirred for overnight at RT. The product was purified by HPLC. R_f_(HPLC): 14.9 min. UV: 352 nm.

*General Procedure for the Synthesis of Compounds* **41** *and* **42**. A mixture of compound **14** or **15** (1.00 mmol), and compound **35** (0.22 g, 0.84 mmol; [28]) in dry TEA (5 mL) and THF (10 mL) was de-aerated with argon. After an addition of bis(triphenylphosphine)palladium(II) dichloride (10 mg, 14 μmol) and CuI (6 mg, 28 μmol), the mixture was stirred overnight at 55 °C and evaporated to dryness. The residue was dissolved in CH_2_Cl_2_ (30 mL) washed with H_2_O (3 × 10 mL), dried with Na_2_SO_4_ and evaporated to dryness. The product was purified by silica gel column chromatography.

*Diethyl 2,2′-{{4-[(2-(ethoxycarbonyl)-6-(hydroxymethyl)pyridin-4-yl]ethynyl)-1,3-phenylene}bis-(oxy)}-di-acetate* (**41**). The product was purified by silica gel column chromatography using MeOH/CH_2_Cl_2_ (5:95) as an eluent. Yield: 80%. ^1^H-NMR (CDCl_3,_ δ ppm): 8.08 (2H, s), 7.60 (2H, s), 7.45 (1H, d, *J* = 8.5 Hz), 6.50 (1H, dd, *J* = 2.0 and 8.5 Hz), 6.45 (1H, d, *J* = 2.0 Hz), 4.85 (2H, s), 4.71 (2H, s), 4.63 (2H, s), 4.47 (2H, q, *J* = 7.1 Hz), 4.30 (2H, q, *J* = 7.1 Hz), 4.29 (2H, q, *J* = 7.1 Hz), 1.44 (3H, t, *J* = 7.1 Hz), 1.32 (3H, t, *J* = 7.1 Hz), 1.31 (3H, t, *J* = 7.1 Hz). ^13^C-NMR (CDCl_3_, δ ppm): 168.11, 168.00, 164.63, 161.71, 160.08, 159.95, 147.27, 134.81, 127.02, 125.50, 106.42, 105.38, 100.99, 91.30, 89.67, 65.91, 65.34, 64.32, 62.24, 61.93, 61.54, 14.20, 14.19, 14.07. MS(ESI-TOF) calculated for C_25_H_27_NO_9_[M + H]^+^ 486.18; found 486.46.

*Triethyl 2,2′,2′′-{{2-{[2-(ethoxycarbonyl)-6-(hydroxymethyl)pyridin-4-yl]ethynyl)}benzene-1,3,5-triyl}tris-(oxy)}triacetate* (**42**). The product was purified by silica gel column chromatography using EtOAc/petroleum ether/TEA (from 69:30:1 to 89:10:1) as an eluent. Yield: 76%. ^1^H-NMR (CDCl_3,_ δ ppm): 8.11 (1H, d, *J* = 0.5 Hz), 7.64 (1H, d, *J* = 0.5 Hz), 6.09 (2H, s), 4.84 (2 H, s), 4.70 (4H, s), 4.58 (2H, s), 4.47 (2H, q, *J* = 7.1 Hz), 4.29 (4H, q, *J* = 7.1 Hz), 4.28 (2 H, q, *J* = 7.1 Hz), 3.45 (1 H, bs), 1.44 (3H, t, *J* = 7.1 Hz), 1.32 (3H, t, *J* = 7.1 Hz), 1.31 (6H, t, *J* = 7.1 Hz). ^13^C-NMR (CDCl3, δ ppm): 167.97, 167.89, 164.70, 161.04, 160.22, 158.87, 147.21, 134.13, 125.42, 125.17, 96.17, 94.27, 93.80, 87.78, 66.21, 65.42, 64.29, 61.86, 61.61, 61.49, 14.20, 14.08, 14.06. MS(ESI-TOF) calculated for C_29_H_33_NO_12_[M + H]^+^ 588.56; found 589.03.

*General Procedure for the Synthesis of Compounds* **43** *and* **44**. A mixture of compound **41** or **42** (0.613 mmol) and PBr_3_ (86 μL, 0.919 mmol) in dry CHCl_3_ (20 mL) was stirred for 2.5 h at RT, neutralized with aqueous 5% NaHCO_3_ (20 mL), the aqueous phase was extracted with CHCl_3_ (2 × 20 mL) and the combined organic phases were dried with Na_2_SO_4_. The product was purified by silica gel column chromatography.

*Diethyl 2,2′-{{4-{[2-(bromomethyl)-6-(ethoxycarbonyl)pyridin-4-yl]ethynyl}-1,3-phenylene}bis-(oxy)}diacetate* (**43**). The product was purified by silica gel column chromatography using EtOH/CH_2_Cl_2_ (10:90) as an eluent. Yield: 89%. ^1^H-NMR (CDCl_3,_ δ ppm): 8.10 (1H, d, *J* = 1.2 Hz), 7.75 (1H, d, *J* = 1.2 Hz), 7.46 (1H, d, *J* = 8.5 Hz), 6.50 (1H, dd, *J* = 2.3 and 8.5 Hz), 6.46 (1H, d, *J* = 2.3 Hz), 4.72 (2H, s), 4.63 (2H, s), 4.60 (2H, s), 4.49 (2H, q, *J* = 7.1 Hz), 4.30 (2H, q, *J* = 7.1 Hz), 4.29 (2H, q, *J* = 7.1 Hz), 1.45 (3H, t, *J* = 7.1 Hz), 1.32 (3H, t, *J* = 7.1 Hz), 1.31 (3H, t, *J* = 7.1 Hz). ^13^C-NMR (CDCl_3,_ δ ppm): 168.10, 167.97, 164.43, 160.05, 160.03, 157.41, 147.95, 134.88, 127.65, 126.00, 106.42, 105.28, 100.97, 91.97, 89.34, 65.92, 65.35, 62.40, 62.09, 61.54, 61.49, 32.88, 14.22, 14.09, 14.07. MS(ESI-TOF) calculated for C_25_H_26_BrNO_8_[M + H]^+^ 548.09 and 550.09; found 548.83 and 550.80.

*Triethyl 2,2′,2′′-{{2-{[2-(bromomethyl)-6-(ethoxycarbonyl)pyridin-4-yl]ethynyl}benzene-1,3,5-triyl}-tris(oxy)}-triacetate* (**44**). The product was purified by silica gel column chromatography using EtOH/CH_2_Cl_2_ (10:90) as an eluent using EtOH/CH_2_Cl_2_ (10:90) as an eluent. Yield: 82%. ^1^H-NMR (CDCl_3,_ δ ppm): 8.12 (1H, d, *J* = 1.3 Hz), 7.79 (1H, d, *J* = 1.3 Hz), 6.08 (2H, s), 4.71 (4H, s), 4.59 (2H, s), 4.51 (2H, s), 4.48 (2H, q, *J* = 7.1 Hz), 4.30 (4H, q, *J* = 7.1 Hz), 4.29 (2H, q, *J* = 7.1 Hz), 1.43 (3H, t, *J* = 7.1 Hz), 1.31 (9 H, t, *J* = 7.1 Hz). ^13^C-NMR (CDCl_3,_ δ ppm): 167.92, 167.79, 164.52, 156.29, 133.27, 125.98, 125.08, 96.09, 93.92, 93.76, 88.21, 66.22, 65.59, 65.43, 62.04, 61.62, 61.51, 32.96, 14.22, 14.07, 14.03. MS(ESI-TOF) calculated for C_29_H_32_BrNO_11_[M + H]^+^ 650.13 and 652.13; found 651.08 and, 653.02.

*Compound* **45**. A mixture of compound **37** (0.41 g, 0.90 mmol), diBoc-TACN (0.14 g, 0.82 mmol), dry K_2_CO_3_ (0.23 g, 1.62 mmol) and dry MeCN (8 mL) was stirred for 24 h at RT. After filtration and washing the solid material with CH_2_Cl_2_, the filtrate was evaporated to dryness. The product was purified by silica gel column chromatography using EtOH/CH_2_Cl_2_ (from 1:99 to 3:97) as an eluent. Yield: 0.31 g (53%). ^1^H-NMR (DMSO-*d*_6_, δ ppm): 11.48 (1H, s), 7.97 (1 H, s), 7.78–7.85 (3H, m), 7.66 (2H, d, *J* = 8.3 Hz), 4.38 (2H, q, *J* = 7.1 Hz); 3.80–3.85 (2H, m), 3.10–3.45 (8H, m), 2.65–2.75 (2H, m), 2.65–2.55 (2H, m), 1.43 (3H, s), 1.42 (3H, s), 1.40 (6H, s), 1.39 (6H, s), 1.34 (3 H, t, *J* = 7.1 Hz). ^13^C-NMR (DMSO-*d*_6_, δ ppm): 164.09, 155.78, 154.96, 154.80, 154.70, 154.56, 154.37, 154.08, 147.22, 137.51, 132.74, 132.54, 129.33, 127.03, 124.69, 120.85, 118.97, 116.69, 114.39, 112.62, 93.89, 86.35, 78.71, 61.44, 61.29, 51.42, 50.18, 49,69, 28.03, 14.02. Both spectra indicate the existence of rigid compound having different structural isomers. MS(ESI-TOF) calculated for C_35_H_44_F_3_N_5_O_7_[M + H]^+^ 704.33; found 705.09.

*Compound* **46**. A mixture of compound **45** (0.29 g, 0.41 mmol) and TFA (2 mL) was stirred for 2 h at RT, evaporated to dryness and triturated with Et_2_O (40 mL). The product (0.34 g, 89%) was centrifuged, washed with Et_2_O (2 × 15 mL) and dried. ^1^H-NMR (DMSO-*d*_6_, δ ppm): 11.54 (1H, s), 7.82 (2 H, d, *J* = 8.4 Hz), 7.81 (1 H, s), 7.80 (1H, s), 7.71 (2 H, d, *J* = 8.4 Hz), 4.44 (2H, q, *J* = 7.0 Hz), 4.17 (2H, s), 3.69 (4H, bs), 3,26 (4H, bs), 2.97 (4H, bs), 1.39 (3H, t, *J* = 7.0 Hz). ^13^C-NMR (DMSO-*d*_6_, δ ppm): 154.41, 160.60, 155.48, 155.18, 154.89, 154.59, 147.17, 138.30, 133.24, 133.06, 129.87, 128.40, 125.22, 121.41, 118,57, 116.19, 114.84, 112.54, 95.54, 86.36, 62.47, 57.68, 50.42, 45.90, 45.36, 14,45. MS(ESI-TOF): calculated C_25_H_28_F_3_N_5_O_3_ [M + 2H]^+^ 505.54; found 505.31.

*General Procedure for the Synthesis of Ligand Esters* **47** *and* **48**. A mixture of compound **46** (0.12 g, 0.15 mmol), **43** or **44** (0.32 mmol), di-isopropylethylamine (0.40 mL) and dry MeCN (3.0 mL) was stirred for 5.5 h at RT and evaporated to dryness. The product was purified by silica gel column chromatography.

*Ligand Ester* **47**. The product was purified by silica gel column chromatography using EtOH/CH_2_Cl_2_/TEA (from 10:90:0 to 10:89:1) as an eluent. Yield: 84%. As the product contains 2–3 rigid isomers, the NMR spectra were too complicated to assign the isomers. MS(ESI-TOF) calculated for C_75_H_78_F_3_N_7_O_19_ [M + H]^+^ 1438.54; found 1439.41.

*Ligand Ester* **48**. The product was purified by silica gel column chromatography using EtOH/CH_2_Cl_2_/TEA (from 10:90:0 to 15:80:5) as an eluent. Yield: 79%. As the product contains 2–3 rigid isomers, the NMR spectra were too complicated to assign the isomers. MS(ESI-TOF): calculated [M + H]^+^1642.60; found 1643.57.

*General Procedure for the Synthesis of Eu(III) Chelates* **49** *and* **50**. A mixture of the compound **47** or **48** (64 μmol) and 0.5 M KOH in EtOH (6.5 mL) was stirred for 1 h at RT and H_2_O (3 mL) was added. After stirring for 4 h at RT, EtOH was evaporated, some H_2_O (2 mL) added and the residue was stirred for 24 h at RT. After addition of citric acid (41 mg, 0.21 mmol) in H_2_O (0.25 mL), the pH was adjusted to ca. 6.5 with 6 M HCl. Europium(III) chloride (26 mg, 71 μmol) in H_2_O (0.25 mL) was added within 10 min and the pH was adjusted to ca. 9.5 with 1 M NaOH. The mixture was stirred for 4–6 weeks at 95 °C (after the analytical HPLC chromatogram showed completed complexation), the pH was adjusted to ca. 7.0 with 1 M HCl, evaporated to dryness, dissolved in 20 mmol Triethylammonium acetate (TEAA) buffer (1 mL) and purified with HPLC. 

*Eu(III) Chelate* **49**. R_f_(HPLC) = 19.2 min. UV: 350 nm. MS(ESI-TOF) calculated for C_59_H_48_EuN_7_O_18_ [M + H]^+^ 1296.24; found 1295.85. Ligand isomers shown in the HPLC during the Eu(III) loading: (1) R_f_(HPLC) = 21.7 min, UV: 347 nm; (2) R_f_(HPLC) = 22.3 min, UV: 339 nm; (3) R_f_(HPLC) = 23.6 min, UV: 343 nm. All of these peaks finally gave the product peak at R_f_(HPLC) = 19.2 min. UV: 350 nm.

*Eu(III) Chelate* **50**. R_f_(HPLC) = 16.0 min. UV: 360 nm MS(ESI-TOF) calculated for C_63_H_62_EuN_7_O_24_ [M + H]^+^ 1444.24; found 1443.96. Ligand isomers shown in HPLC during the Eu(III) loading: (1) R_f_(HPLC) = 18.5 min, UV: 345 nm; (2) R_f_(HPLC) = 20.4 min, UV: 347 nm; (3) R_f_(HPLC) = 21.7 min, UV: 347 nm. All of these peaks finally gave the product peak at R_f_ = 16.0 min, UV = 360 nm. 

The Eu complex formation caused the observed bathochromic shift of 13–15 nm at UV. This was separately secured with the complex **50** by an additional HPLC purification of the ligand isomers and loadings of Eu(III) ion to each isomers. All of these tests gave finally the same product at R_f_(HPLC) = 16.0 min.

*General Procedure for the Synthesis of Eu(III) Chelates* **7** *and* **8**. An aqueous solution (1 mL) of the chelate **49** or **50** (21 μmol) was added within 5 min to a mixture of CSCl_2_ (22 µL, 0.29 mmol) and NaHCO_3_ (28 mg, 0.33 mmol) and CHCl_3_ (1 mL). After stirring for 40 min at RT, the two phases were separated and the aqueous phase was washed with CHCl_3_ (3 × 1 mL). The product was precipitated with acetone (ca. 45 mL), isolated by centrifugation, washed with acetone (2 × 10 mL), and dried overnight in a vacuum desiccator. The products were used as such for next phase or for labeling the antibodies.

*Eu(III) Chelate* **7**. R_f_(HPLC) = 19.9 min. UV: 350 nm.

*Eu(III) Chelate* **8**. R_f_(HPLC) = 17.6 min. UV: 356 nm.

*General Procedure for the Synthesis of Eu(III) Chelates* **7a** *and* **8a**. A mixture of chelate **7** or **8** (2 mg) and taurine (2 mg) in 50 mM Na_2_CO_3_ buffer (300 μL, pH 9.8) was stirred overnight at RT. The product was purified by using HPLC. 

*Eu(III) Chelate* **7a**. R_f_(HPLC) = 17.2 min. UV = 348 nm.

*Eu(III) Chelate* **8a**. Rf(HPLC) = 15.2 min. UV = 354 nm.

### 3.3. Coupling of the Chelates ***2***–***8*** to Protein

The activated chelates were coupled to a troponin I antibody with a similar method as described in [15] by using 100–300 fold excess of the labelling reagents. The reagents were incubated with the antibody (1 mg) in 100–350 mM carbonate buffer (500–1000 μL, pH 9.8) overnight at RT. Labeled antibody was separated from the chelate excess on a Superdex 200 GL 10/30 gel filtration column (GE Healthcare Life Sciences) by using TRIS buffer (TRIS 50 mM, NaCl 0.9%, pH 7.75) as an eluent. The fractions containing the antibody were pooled and the europium concentration was measured by UV and confirmed by ICP-MS to obtain the labeling degrees i.e., Eu content per antibody. Finally, bovine serum albumin was added to a concentration of 1g/L to the solutions containing the europium labeled antibodies. The solutions were stored at 4 °C.

### 3.4. Fluorescence Measurements of the Eu Chelates ***2a***–***8a*** and the Antibody Conjugates (***2b***–***8b***)

The fluorescence parameters for the Eu Chelates **2a**–**8a** as well as the labeled antibodies (**2b**–**8b**) were analyzed in 50 mM TRIS buffer (pH 7.75) and the Eu concentrations were measured by using an ICP-MS (inductively coupled plasma mass spectrometer) instrument, ELAN 6100 DRC Plus (PerkinElmer, Waltham, MA, USA), in quantitative mode. The analyzing parameters were: the Peak Hopping mode, 20 sweeps/reading, 7 replicates, the Dwell time and the integration time was 50 ms and 1000 ms, respectively. Rhodium was used as the internal standard and the europium was measured on Mass 152.929. A commercial multi-standard IMS–101, ICP-MS calibration standard 1 (ULTRA Scientific, North Kingstown, RI, USA) was used for the calibration. The sample preparation for the ICP-MS was done by using a digestion procedure i.e., the Microwave Sample preparation System, Multiwave 3000 (Anton Paar GmbH, Graz, Austria). The Eu chelate in the 50 mM TRIS buffer was digested with microwave in mixture of Suprapur acids, HNO_3_ (5 mL) and H_2_O_2_ (1 mL). Afterwards, the sample was diluted with deionized water (100 mL).

The fluorescence measurements were standardized using the Chelate **1a** and **1b** as a reference. The photochemical values are based on earlier measurements [15,20]. The emission intensities of the solutions were measured using the most intense emission line at 613–618 nm. Fluorescence efficiencies and decay times were determined with VICTOR^TM^ X4 multilabel reader (PerkinElmer) and Cary Eclipse spectrofluorometer (Agilent Technologies, Santa Clara, CA, USA), respectively.

### 3.5. Immunoassay and Signal Measurement from the Dried Microwell Surface

The troponin I antibody labeled with the chelate **2b**–**8b** was tested in sandwich immunoassay for cardiac troponin I. A troponin I antibody labelled with **1b** was used as a reference compound.

General assay procedure: 10 µL of diluted tracer antibody (5 ng/µL) and 20 µL of troponin I standard solution were pipetted to a pre-coated assay well (single wells in 96 well plate format, wells coated with streptavidin and a biotinylated capture antibody against troponin I, Radiometer Turku Oy). The reaction mixtures were incubated 20 min at 36 °C with shaking. The wells were washed 6 times and dried prior to measurement with VICTOR Plate fluorometer. More exact details have been described in literature [15,20].

## 4. Conclusions

Altogether, seven new Eu(III) labeling chelates having two to three 4-(phenylethynyl)pyridine chromophors in three different basic structural designs were synthesized and their key photophysical properties were studied in this work. The focus of the study was to improve the signal level of the label currently used in immunoassays, and, thus, to clarify the possibilities to enhance the assay sensitivity. The significance of ligand antennas ILCT state as an alternative sensitization process on the label’s brightness has been demonstrated, and the enhanced luminescence intensities were unprecedentedly large especially when the signal was measured on the dried surface. These new labeling chelates provide a solution to improve the assay sensitivity up-to tenfold from the present concepts.

## Figures and Tables

**Table 1:** Absorption maxima (λ_abs_) and excitation maxima (λ_exc_), luminescence decay times (τ), molar absorption coefficients (ε), brightness (εΦ) and quantum yields (Φ) in TRIS buffer (pH 7.75) as well as the enhanced brightness on dried surface.

Chelate	λ_abs_ (nm)	λ_exc_ (nm)	τ (ms)	ε (M^−1 ^cm^−1^)	εΦ (M^−1 ^cm^−1^)	Φ (%)	εΦ ^1^ (M^−1^ cm^−1^)
1a	320	325	1.01	55,000	4800	9	-
1b	325	325	1.01	55,000	4800	9	8000
2a	343	340	0.96	57,000	8500	15	-
2b	344	340	0.97	55,000	5700	10	16,100
3a	351	346	0.88	58,000	4400	8	-
3b	349	345	0.90	68,000	4900	7	20,200
4a	343	343	0.84	85,000	14,900	18	-
4b	348	343	0.80	77,000	16,100	21	24,100
5a	349	343	0.70	64,000	16,400	26	-
5b	351	344	0.69	78,000	11,400	15	34,400
6a	352	342	0.77	80,000	15,400	19	-
6b	350	345	0.69	75,000	9700	13	37,200
7a	348	341	0.58	89,000	600	0.7	-
7b	352	346	0.51	99,000	500	0.5	40,200
8a	354	345	0.48	77,000	700	0.9	-
8b	356	348	0.42	104,000	1000	1.0	69,500

^1^ Enhanced brightness after drying.
